# Elevation of cell-associated HIV-1 transcripts in CSF CD4+ T cells, despite effective antiretroviral therapy, is linked to brain injury

**DOI:** 10.1073/pnas.2210584119

**Published:** 2022-11-21

**Authors:** Kazuo Suzuki, John Zaunders, Thomas M. Gates, Angelique Levert, Shannen Butterly, Zhixin Liu, Takaomi Ishida, Sarah Palmer, Caroline D. Rae, Lauriane Jugé, Lucette A. Cysique, Bruce J. Brew

**Affiliations:** ^a^New South Wales State Reference Laboratory for HIV, Centre for Applied Medical Research, St Vincent's Hospital, Sydney, NSW 2010, Australia; ^b^St Vincent’s Clinical School, Faculty of Medicine, University of New South Wales, Sydney, NSW 2010, Australia; ^c^Clinical Research Program, Centre for Applied Medical Research, St Vincent’s Hospital, Sydney, NSW 2010, Australia; ^d^Department of Neurology and Immunology, St Vincent’s Hospital, Sydney, NSW 2010, Australia; ^e^Peter Duncan Neurosciences Unit, Centre for Applied Medical Research, St Vincent’s Hospital, Sydney, NSW 2010, Australia; ^f^School of Psychology, Faculty of Science, University of New South Wales, Sydney 2054, Australia; ^g^Stats Central, University of New South Wales, Sydney 2052, Australia; ^h^Denka Co. Ltd., Tokyo 103-8338, Japan; ^i^Westmead Institute for Medical Research, University of Sydney, Sydney, NSW 2145, Australia; ^j^Neuroscience Research Australia, Sydney, NSW 2145, Australia; ^k^Faculty of Medicine, University of New South Wales, Sydney 2052, Australia; ^l^School of Medicine, University of Notre Dame, Sydney, NSW 2010, Australia

**Keywords:** cerebrospinal fluid, neuropathogenesis, intracellular HIV-1 RNA-transcripts, CD4+T cells, brain injury

## Abstract

Antiretroviral therapy (ART) can attain prolonged undetectable HIV-1 in plasma and cerebrospinal fluid (CSF), but brain injury remains prevalent in people living with HIV-1 infection (PLHIV). We investigated cell-associated (CA)-HIV-1 RNA transcripts in cells in CSF and blood, using the highly sensitive Double-R assay, together with proton Magnetic Resonance Spectroscopy (^1^H MRS) of major brain metabolites, in sixteen PLHIV. 14/16 CSF cell samples had quantifiable CA-HIV-1 RNA, at levels significantly higher than in their PBMCs (median 9,266 vs 185 copies /106 CD4+ T-cells; p<0.0001). In individual PLHIV, higher levels of HIV-1 transcripts in CSF cells were associated with greater brain injury in the frontal white matter (Std β=-0.73; p=0.007) and posterior cingulate (Std β=-0.61; p=0.03). 18-colour flow cytometry revealed that the CSF cells were 91% memory T-cells, equally CD4+ and CD8+ T-cells, but fewer B cells (0.4 %), and monocytes (3.1%). CXCR3^+^CD49d^+^integrin β7-, CCR5^+^CD4^+^ T-cells were highly enriched in CSF, compared with PBMC (p <0.001). However, CA-HIV-1 RNA could not be detected in 10/16 preparations of highly purified monocytes from PBMC, and was extremely low in the other six. Our data show that elevated HIV-1 transcripts in CSF cells were associated with brain injury, despite suppressive ART. The cellular source is most likely memory CD4^+^ T cells from blood, rather than trafficking monocytes. Future research should focus on inhibitors of this transcription to reduce local production of potentially neurotoxic and inflammatory viral products.

## Significance

Despite antiretroviral therapy of HIV infection, and effective viral suppression, we found unexpectedly high levels of intracellular HIV RNA transcription in cerebrospinal fluid cells, which correlated with ongoing brain injury. These results challenge prevalent concepts that HIV neuropathogenesis is the result of legacy damage from the past or current injury from comorbidities. Current antiretroviral drugs do not inhibit the transcription stage of the HIV life cycle. Thus, brain injury results from an active HIV process despite suppressing spreading viral infection. Further, the monocyte HIV transcript burden was vastly less than in CD4 T cells, thereby diminishing the classically held belief that circulating infected monocytes are the cause of HIV neuropathogenesis. The results highlight the need for novel drugs that target transcription.

Despite suppressive antiretroviral therapy (ART) leading to undetectable HIV-1 RNA in both plasma and cerebrospinal fluid (CSF), in vivo brain injury in people living with chronic HIV-1 infection (PLHIV), revealed by proton magnetic resonance spectroscopy (^1^H MRS) and neuropsychological testing, persists and remains common (1).

The mechanisms underlying this persistent brain injury remain unclear. Indeed, in chronic HIV-1 infection, concurrent mechanisms of brain injury may include neurological and psychiatric confounds, age-related comorbidities, the legacy effect of pre-ART deficit, and poor brain penetration of ART, but also possible ART toxicities (2, 3). A direct HIV-1 cause of brain injury in chronic HIV-1 infection under ART has only been discussed in the context of relatively rare occurrences of “CSF escape” where CSF viral load becomes detectable, while plasma viral load remains undetectable (4, 5). Nonetheless, the role of viral reservoirs and sanctuaries cannot be excluded, especially viral replication within the parenchyma of the central nervous system (CNS) (6). Therefore, consideration of brain injury despite suppressive ART should also investigate the reservoir (HIV-1 DNA copy number), its location within or outside the CNS, its transcriptional activity (cell-associated (CA) HIV-1 RNA copy numbers), and the virus replication competence (production of cell-free virus). The current interpretation of the reservoir and sanctuary as they pertain to the CSF (as an indirect marker of the brain) has been influenced by assay methods that have only detected HIV-1 DNA or RNA in CSF cells at relatively low levels, under fully suppressive ART (7). However, despite these mostly undetectable reservoir levels in CSF cells, very low levels of cell-free virus, typically around 1 copy/ml of CSF, have been reported in 40% of subjects regarded as clinically fully suppressed on ART (8). Taken together, the HIV reservoir size and activity in CSF cells may have been underestimated, suggesting that it has an unappreciated role in HIV neuropathogenesis.

The Double R assay, based on the πCode End-Point PCR platform, is much more sensitive than previous assays at detecting HIV-1 DNA and RNA (9, 10). It can detect the presence of both spliced and unspliced mRNA, which could then be translated into viral proteins such as Tat, Env, Nef, Rev, and Vpr. These are important as they are neurotoxic (3, 11–15) and could still be produced from the reservoir even though most of the reservoir cannot produce replication-competent whole virus (16, 17). Furthermore, suppressive ART does not stop the production of these viral components once HIV-1 is integrated into the host genome; ART targets almost every step of the life cycle of HIV-1 from receptor-mediated entry to budding from the cell surface. However, it does not specifically affect transcription of RNA from the promoter in the integrated HIV-1 proviral DNA. Therefore, transcription and then translation of early viral proteins such as Tat may still occur despite suppressive ART (3). We therefore hypothesized that brain injury is related to the production of these proteins in the context of suppressive ART.

We tested this hypothesis by using the highly sensitive Double R assay to detect and quantify HIV-1 DNA copy number and CA-RNA transcripts in CSF cells and PBMCs from PLHIV on suppressive ART, correlating the findings with brain injury as assessed by ^1^H MRS. We also examined the cellular composition of the CSF to determine the likely source of these transcripts.

## Results

### Sample Characteristics.

The cohort comprised 16 PLHIV (Table 1). They were aged 62.9 y on average, belonged to English-speaking background, and were Australian White men with chronic, treated HIV (median HIV infection duration 28 y, on suppressive ART for a median of 22 y, nine (56.3%) initiated suppressive ART more than 12 mo postseroconversion). Nine (56.3%) had an historical diagnosis of AIDS. The sample was otherwise homogeneous in terms of their global cognitive functioning which was close to or slightly below the normative mean, reflecting some degree of chronic vulnerability. Six participants (37.5%) had an historical diagnosis of HIV-associated neurocognitive disorders (HAND) and five (33.3%) had current mild HAND (1 ANI: Asymptomatic neurocognitive impairment, 4 MND: Mild neurocognitive disorder). The median nadir CD4+ T cell count was 185 cells/µl and the current median CD4+ T cell count was 759 cells/µl.

**Table 1.** Sample demographic and disease characteristics

Demographics	
N	16
Age (years), M (SD)	62.88 (11.84)
Education (years), med (IQR)	13 (13–15)*
Sex: male, n (%)	16 (100%)
Ethnicity: White ESB, n (%)	16 (100%)
*HIV disease characteristics*	
Nadir CD4+ T cell count, med (IQR)	185 (85–327.5)
Historical AIDS, n (%)	9 (56.25%)
HIV infection:	
Duration (years), med (IQR)	28 (12.75–34)
Seroconversion to ART initiation (years), med (IQR)	4.5 (0–10)
ART initiation > 1 y after seroconversion (n, %)	9 (56.25%)
Duration on ART (years), med (IQR)	22 (10.5–27)
CPE Rank Score, med (IQR)	8.5 (8–11.5)^†^
Blood CD4+ T cell count, med (IQR)	758.5 (502.25–980.75)
Blood CD8+ T cell count, med (IQR)	701 (456.75–900.75)
Past HAND, n (%)	6 (37.50%)
Current HAND, n (%)	5 (33.33%)*
HAND status, n (%)	1 (7%) ANI^a^, 4 (27%) MND^b^
GDS, med (IQR)	0.4 (0–0.53)*
Mean NP T-Score, M (SD)	48.31 (4.35)*

^*^n = 15.

^†^CPE (CNS penetration efficiency) Rank Score (18). Notes. The CPE rank score was not associated with any outcomes including brain injury as measured by ^1^H MRS.

^a^ANI: Asymptomatic neurocognitive impairment.

^b^MND: Mild neurocognitive disorder.ESB: English-speaking background, IQR: interquartile range.

### Detection of Cell-Free HIV-1 in CSF and Plasma.

CSF cell-free HIV RNA was <80 copies/ml for all samples, using the Roche Amplicor assay. Using the single-copy assay (SCA), HIV-1 RNA was undetectable in the 13 (81%) CSF samples with sufficient volume available for testing.

Plasma samples were fully virally suppressed with HIV RNA <50 copies/ml using the Roche Amplicor assay. SCA analysis revealed that HIV-1 RNA was undetectable in the plasma of eight samples (50%) (<0.3 copies/ml). In the remaining eight detectable samples, the median was 37 copies/ml.

### Detection of CA HIV-1 in CSF Cells and PBMCs Using the Double R Assay.

**Fig. 1.**
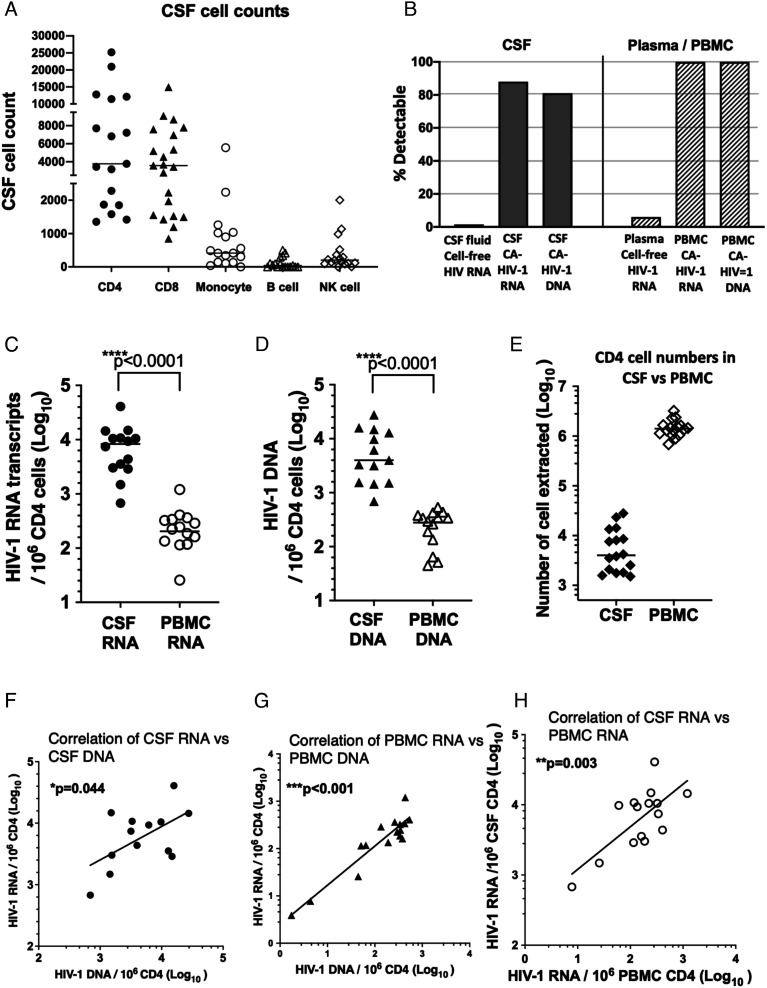
Comparison of CSF and PBMC cell numbers and HIV-1 CA-RNA and DNA levels. (*A*) Range of recovered CSF cell counts for different CD45+ cells obtained in the pellets, including CD3+CD4+ and CD3+CD8+ T lymphocytes, CD14+ monocytes, CD19+ B cells, and CD16+CD56+ NK cells; (*B*) Rate of detectability of cell-free virus using single-copy assay, in CSF and plasma and cell-associated (CA) HIV-1 RNA and DNA in CSF cells and PBMC, respectively; (*C*) comparison of HIV-1 CA-RNA copies, normalized per 10^6^ CD4 T cells, for CSF cells and PBMC; (*D*) comparison of HIV-1 CA-DNA copies, normalized per 10^6^ CD4 T cells, for CSF cells and PBMC; (*E*) numbers of CSF cells and PBMC used for extractions; (*F*) correlation of copy numbers of HIV-1 CA-RNA vs. DNA in CSF cells; (*G*) correlation of copy numbers of HIV-1 CA-RNA vs. DNA in CSF cells; and (*H*) correlation of copy numbers of HIV-1 CA-RNA in CSF cells vs. HIV-1 CA-RNA in PBMC.

The cells in the CSF were dominated by CD3+ T cells, which were evenly divided between CD4+ and CD8+ T cells (*SI Appendix*, Fig. S1*A*). Other cell types in the CSF were proportionally much lower, including monocytes (3.1% of CSF cells; *SI Appendix*, Fig. S1*B*), natural killer (NK) cells (2.0%), and B cells (0.4%). The numbers of these cells recovered in the CSF samples are shown in Fig. 1*A*. In particular, the number of CD4+ T cells (median: 3,605 cells) greatly outnumbered that of monocytes (378 cells), such that, in all but two samples, there was an average >20-fold difference. Furthermore, the monocytes were >90% CD14+CD16+, the phenotype of intermediate monocytes in blood.

These CSF cells contained detectable HIV-1 CA-RNA-transcription copies in 14/16 (88%) participant samples, and HIV-1 CA-DNA copies were detected in CSF cells from 13/16 (81%) participant samples (Fig. 1 *B*, *Left*). In contrast to this finding, the Amplicor and SCA analyses were unable to detect HIV-1 RNA in the cell-free CSF fluid fraction.

In PBMCs, HIV-1 CA-RNA transcripts and HIV-1 CA-DNA copies were both detected in 16/16 (100%) participant samples (Fig. 1 *B*, *Right*). Once again, this contrasted with limited detection of cell-free HIV-1 RNA in plasma, as described above.

The quantitation from the Double R assay was normalized as HIV-1 copy number per 1 × 10^6^ CD4+ T cells, to permit comparison with the corresponding PBMC samples for each participant. The results showed that HIV-1 CA-RNA-transcription levels were significantly higher in CSF cells than those in paired PBMCs (Fig. 1*C*: median 9,266 vs. 185 copies/10^6^ CD4+ T cells; *P* < 0.0001). Total HIV-1 CA-DNA levels were also significantly higher in CSF cells than those of the corresponding PBMCs (Fig. 1*D*: median 4,021 vs. 261 copies/10^6^ CD4+ T cells; *P* < 0.0001). Note that there were 300-fold fewer CD4+ T cells extracted from the CSF cells compared to PBMCs (Fig. 1*E*), demonstrating how enriched the HIV-1 CA-RNA-transcription and HIV-1 CA-DNA levels were in CSF cells.

Consistent with our earlier study of PBMCs from fully suppressed patients (10), there was a medium correlation between HIV-1 CA-RNA transcription and HIV-1 CA-DNA in CSF cells (Fig. 1*F*; r = 0.58; *P* = 0.04). Similarly, there was a medium to large correlation between HIV-1 CA-RNA transcription and HIV-1 CA-DNA in CD4+ T cells from PBMCs (Fig. 1*G*; r = 0.72; *P* < 0.001).

Importantly, there was a highly significant correlation between HIV-1 CA-RNA transcription in CSF cells and HIV-1 CA-RNA transcription in CD4+ T cells from PBMCs (Fig. 1*H*; r = 0.83; *P* = 0.003).

### HIV-1 CA-RNA and HIV-1 CA-DNA Relationships with Brain ^1^H MRS (Fig. 2).

**Fig. 2.**
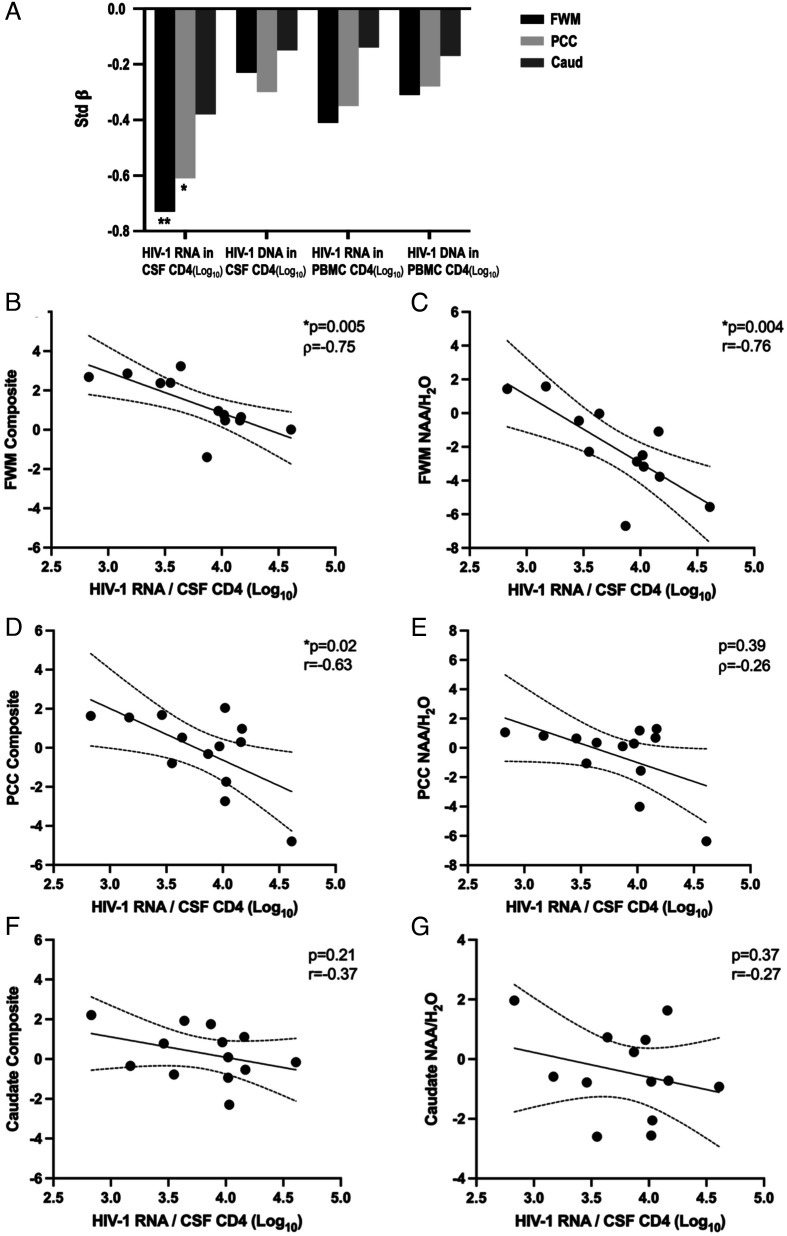
Associations of ^1^H MRS with HIV-1 CA-RNA transcripts and DNA copies. (*A*) HIV-1 CA-RNA and DNA associations with ^1^H MRS voxel composite scores. Univariate associations between log-transformed HIV-1 CA-RNA and DNA copy numbers and age-corrected ^1^H MRS voxel composite z-scores are presented. Asterisk denotes statistical significance at * *P* < 0.05, **P < 0.01. For the posterior cingulate cortex (PCC)/CSF HIV-1 CA-RNA model, addition of early vs. late initiation of suppressive ART at step 2 led to a small decrease in the strength of the association between the PCC composite score and CSF HIV-1 CA-RNA such that the effect was no longer statistically significant (Std β = −0.53, *P* = 0.06), and correlations of HIV-1 CA-RNA in CSF cells with: (*B*) frontal white matter (FWM) composite score; (*C*) FWM NAA/H_2_O; (*D*) PCC composite score; (*E*) PCC NAA/H_2_O; (*F*) Right caudate nucleus composite score; (*G*) Caudate nuclei NAA/H_2_O. Nonparametric or parametric correlations were selected depending on whether the distribution was normal.

HIV-1 CA-RNA transcription in CSF CD4+ T cells showed an inverse relationship of large effect size with the frontal white matter (FWM) (Std β = −0.73, *P* = 0.007) and medium effect size with the posterior cingulate cortex (PCC) 1H MRS composite scores (Std β = −0.61, *P* = 0.03) (*SI Appendix*, Table S2). The univariate association between HIV-1 CA-RNA transcription and the caudate composite score was weaker (Std β = −0.38; *P* = 0.21). The univariate relationships of HIV-1 CA-RNA-transcription levels in PBMC CD4+ T cells with the three ^1^H MRS voxel composites ranged from small to medium in effect size (Std βs = −0.14 to −0.41; *P*s > .15). The associations between HIV-1 CA-DNA levels and the three ^1^H MRS voxel composite scores were nonsignificant and of small effect size (in CSF cells: Std βs = −0.15 to −0.30; *P*s > 0.34; in PBMCs: Std βs = −0.17 to −0.31; *P*s > 0.28).

*N*-acetyl aspartate (NAA) and creatine contributed the most to the three voxel composite MRS scores (*SI Appendix*, Fig. S3). We therefore reconducted the above analysis selecting the NAA/H_2_O ratio (reflecting axonal/neuronal damage) in place of the voxel composite score. Similar to the voxel composite model, a significant large inverse association was observed between FWM NAA/H_2_O and HIV-1 CA-RNA transcription in CSF CD4+ cells (Std β = −0.76; *P* = 0.004) (*SI Appendix*, Table S3). There was also a significant medium-sized inverse relationship between FWM NAA/H_2_O and HIV-1 CA-RNA transcription in PBMC CD4+ cells (Std β = −0.76; *P* = 0.004). Inverse relationships of similar magnitude were also observed between NAA/H_2_O in the PCC and HIV-1 CA-RNA transcription in CSF CD4+ cells (Std β = −0.53; *P* = 0.06), as well as between NAA/H_2_O in the FWM and HIV-1 CA-DNA in PBMC CD4+ T cells (Std β = −0.51; *P* = 0.06), although these effects only reached marginal statistical significance.

### HIV-1 CA-RNA and HIV-1 CA-DNA Relationships with Time to ART Initiation and HAND Status.

We note that the FWM composite score did not differ between early and late ART initiation (rho = −0.10; *P* = 0.72); the PCC composite score did not differ between early and late ART initiation (rho = −0.25; *P* = 0.36); the caudate composite score did not differ between early and late ART initiation (Rho = 0.34; *P* = 0.21, in this case, early treated participant tended to have worse brain injury). Nevertheless, to encompass the treatment dimension that is commonly varied in people with long-term HIV infection, we developed a regression model correcting for this effect. This was also done because of the association between ART timing and HIV-1 CA-DNA, delineating the larger reservoir in those initiated late. The regimen for each participant is provided in *SI Appendix*, Table S4.

Indeed, at step 2 of the hierarchical linear regression models, late initiation of suppressive ART (i.e., >12 mo post-seroconversion) was consistently associated with HIV-1 CA-DNA in CSF and PBMC CD4+ T cells and HIV-1 CA-RNA levels, although to a lower level than early initiation of ART (*SI Appendix*, Table S3). Furthermore, this effect was strongest in the models focused on PBMCs, reaching a medium effect size (Std βs = 0.52–0.69; *P*s < 0.05). Similar effect sizes were observed in caudate-based models focusing on CSF HIV-1 CA-DNA and RNA (both Std βs = 0.62; *P*s = 0.02–0.06), while smaller (nonsignificant) effects were observed in FWM- and PCC-based models focused on CSF HIV-1 CA-DNA and RNA (Std βs = 0.27–0.47; *P*s > 0.14).

When evaluating suppressive ART exposure as a continuous variable, trends in the same direction emerged, albeit not to a statistically significant level. Specifically, longer time from seroconversion to suppressive ART initiation showed small- to medium-sized associations with higher HIV-1 CA-RNA-transcription levels (ρ = 0.25, *P* = 0.39) and CA-DNA (ρ = 0.47, *P* = 0.11) in CSF CD4+ cells. In contrast, length of time on ART was not associated with HIV-1 CA-RNA transcription (ρ = 0.18, *P* = 0.53) or HIV-1 CA-DNA in CSF cells (ρ = −0.02, *P* = 0.95).

We failed to detect HIV-1 CA-RNA transcripts in CSF cells from two participants, both of whom had current mild HAND, and were unable to detect HIV-1 CA-DNA in CSF cells from three patients, two of whom had current mild HAND. This affected our capacity to investigate the association between the Double R assay outcomes and HAND status in the current sample. (See additional neuropsychological results in *SI Appendix*.)

### Source of HIV-1 RNA Transcripts in CSF Cells.

**Fig. 3.**
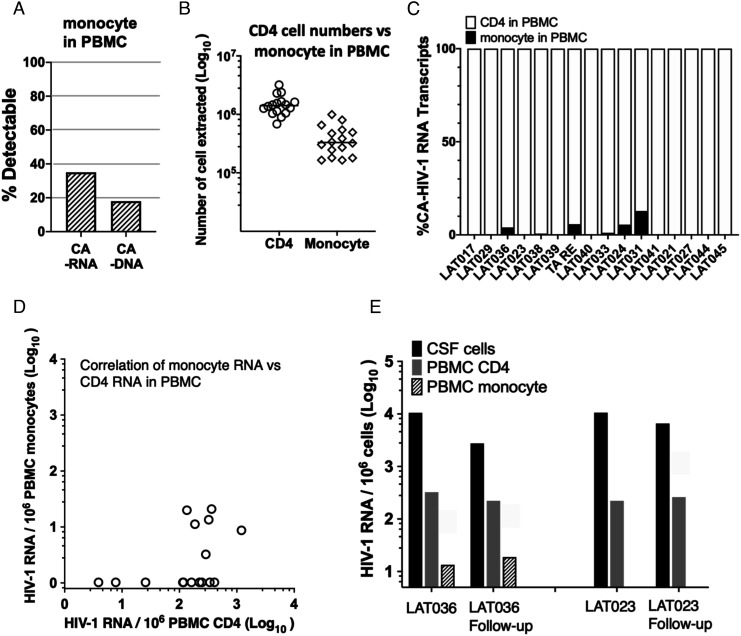
Comparison of CA HIV-1 RNA transcripts in CD4^+^T cells and monocytes in PBMCs. (*A*) Rate of detectability of cell-associated (CA) HIV-1 RNA and DNA in highly purified CD14+ monocytes from PBMC; (*B*) number of highly purified CD14+ monocytes and number of CD4+ T cells from CD14-neg PBMC used for extractions for the Double R assay; (*C*) relative contribution of CD4 T cells and CD14+ monocytes to the total number of CA HIV-1 RNA transcripts from PBMC; (*D*) comparison of CA HIV-1 RNA copy numbers in CD14+ monocytes from PBMC vs. CD4 T cells in PBMC, in individual patient samples; (*E*) consistent results for CA HIV-1 RNA from CSF cells, PBMC CD4 T cells, and PBMC CD14+ monocytes, for longitudinal follow-up samples from two participants.

It is widely believed that monocyte/macrophage lineage cells are a major target cell for HIV-1 infection in the CNS, particularly associated with cognitive impairment (19). As the number of monocytes in the current CSF samples was so low, we instead purified monocytes from PBMC to study, using the Double R assay, whether they contained HIV-1 RNA transcripts or HIV-1 DNA. Fig. 3*A* shows that only 6/16 (35%) participants’ purified monocytes from PBMC had detectable HIV-1 CA-RNA transcripts and only 3/16 (18%) participants had detectable HIV-1 CA-DNA in blood monocytes.

The numbers of monocytes isolated from PBMC that were used to extract RNA and DNA are shown in Fig. 3*B*. It should be noted that the number used was approximately 1,000 times higher than the number of monocytes observed in the CSF samples (Fig. 1*A*), yet HIV-1 RNA transcripts were still not detected in 10/16 monocyte preparations from PBMC, clearly not comparable to the detectability in CSF cells. It is also important to note that we carefully monitored the number of contaminating CD4+ T cells in the monocyte preparations, with a median of 0.26% (IQR: 0.19–0.35%).

Furthermore, the calculated contribution of monocytes to HIV-1 CA-RNA transcripts in PBMCs is shown in Fig. 3*C*, and is extremely low, even in the minority of cases where monocytes had detectable transcripts. The direct comparison of transcript numbers in monocytes vs. those in CD4+ T cells from the same PMBC samples shows the extremely low level of HIV-1 CA-RNA transcripts in monocytes relative to CD4+ T cells, when normalized to 1 × 10^6^ cells (Fig. 3*D*).

For two participants, there were collected CSF/PBMC paired samples on two occasions each, 6 mo apart. The results showed that HIV-1 CA-RNA transcripts in CSF cells, PBMC CD4+ T cells, and monocytes were maintained at very similar levels over this period (Fig. 3*E*)

### Comparative Immunological Profiles of CD4+ T Cells in CSF Cells and in PBMCs.

A very high proportion of the cells in CSF were memory (CD45RA negative) CD4+ T cells and expressed the C-X-C motif chemokine receptor 3 (CXCR3) and the integrin alpha 4 (also known as CD49d), as shown in the representative flow plot, upper left of *SI Appendix*, Fig. S2, but lacked the integrin ß7 (upper flow plot, second from left, *SI Appendix*, Fig. S2). Furthermore, a majority of the CD4+ T cells expressed the HIV coreceptor CC chemokine receptor 5 (CCR5) (upper flow plot, third from left, *SI Appendix*, Fig. S2) and expressed the activation markers CD38 and HLA-DR, either singly or in combination (upper flow plot, far right, *SI Appendix*, Fig. S2).

**Fig. 4.**
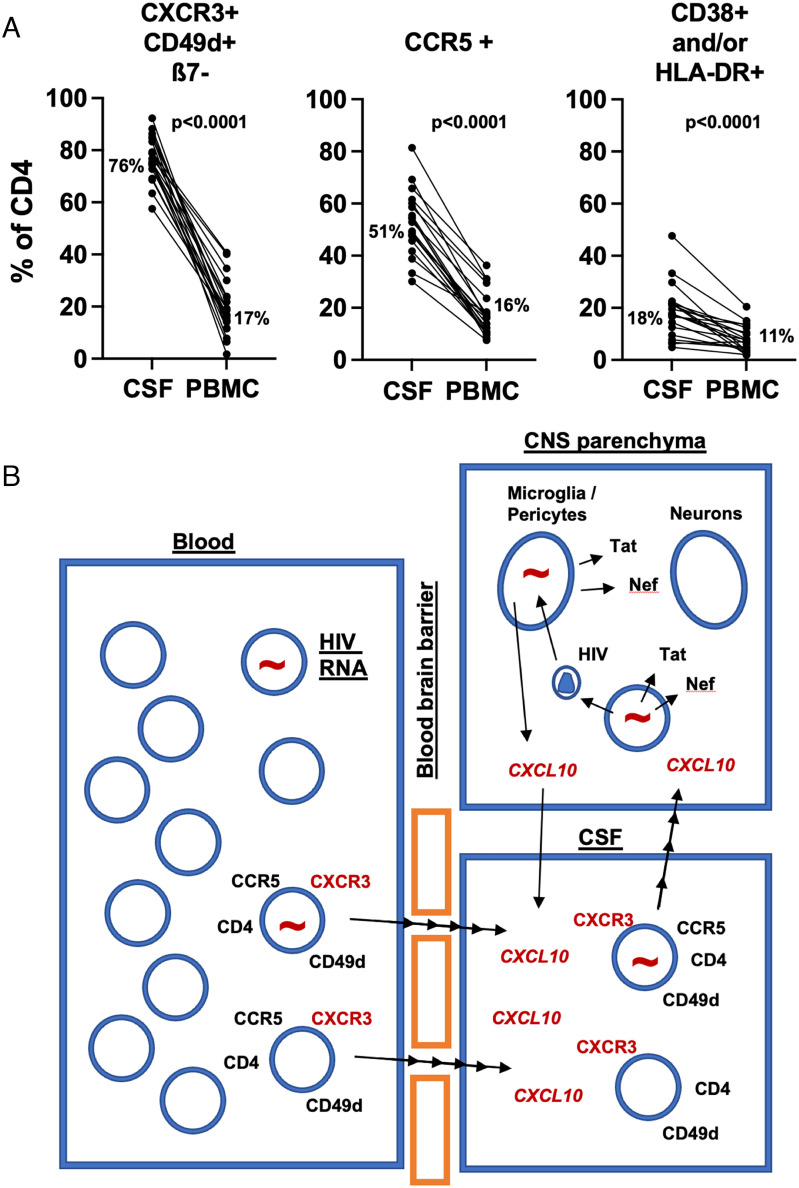
Memory CD4+ T cell subsets in CSF cells vs. PBMCs. (*A*) comparisons of % of CXCR3+CD49d+ß7-negative cells in CD4 T cells from CSF vs. PBMC; % of CCR5+ cells in CD4 T cells from CSF vs. PBMC; and % of combined CD38+ and/or HLA-DR+ cells in CD4 T cells from CSF vs. PBMC, and (*B*) proposed model of directed migration of CXCR3+ CD4+ T cells from blood, including HIV-infected cells, into CSF and CNS parenchyma, due to elevated concentrations of CXCL10 (IP-10), which are in turn due to detection of HIV RNA and viral proteins by myeloid cells in the brain parenchyma.

By comparison, the corresponding subsets, particularly the CXCR3+CD49d+ memory CD4+ T cells, were at a much lower proportion of CD4+ T cells in the paired PBMC samples, as shown in representative flow plots, lower row, in *SI Appendix*, Fig. S2. These differences, shown in Fig. 4*A*, were highly significant by paired Wilcoxon nonparametric comparisons (*P* < 0.001 for the three comparisons; Fig. 4*A*).

Altogether, in Fig. 4*B*, we propose a model of trafficking of infected CXCR3+CD49d+integrin ß7-negative CCR5+ CD4+ T cells from the circulation into the CSF, as discussed in detail below.

## Discussion

Using a highly sensitive HIV-1 assay, we found seven important results: i) the vast majority of samples of CSF cells and all samples of PBMCs contained HIV-1 CA-RNA and DNA despite undetectable cell-free HIV-1 RNA by other assays of viral load, including the single-copy assay; ii) the levels per cell of HIV-1 CA-RNA and DNA were significantly greater in the CSF than those in PBMCs; iii) CSF HIV-1 CA-RNA levels were associated with in vivo brain injury in the FWM, PCC, and, to a lesser extent, the caudate area -this was driven by reduced neuronal/axonal integrity, as reflected by lower NAA/H_2_O in the FWM and other brain regions; iv) the correlation between HIV-1 CA-RNA in CSF and PBMCs was of a large effect size; v) the cellular composition of the CSF target cells was dominantly CD4+ T cells of the CXCR3+ CCR5+ CD49d+ integrin ß7- (nongut homing) phenotype; there were relatively few monocytes; and vi) the source of HIV-1 CA-RNA and DNA in CSF cells was most likely trafficking of infected CXCR3+ CCR5+ CD4+ T cells from peripheral blood; and vii) HIV DNA or HIV CA-RNA were barely detectable in monocytes in peripheral blood, so that they and other cell types, apart from CD4+ T cells, were most likely not involved in the traffic of infected cells into the CNS.

It is important to emphasize that the participants in our study all had well-controlled HIV infection for many years, through individual optimization of their treatment, to maintain full viral suppression in both plasma and CSF and minimize side effects. Despite this, we still readily detected ongoing HIV transcriptional activity in CSF cells, as we have previously described for CD4+ T cells in PBMCs (10).

Our finding of an elevated HIV-1 reservoir and its activity in CSF cells, despite undetectable cell-free HIV-1 RNA in CSF, confirms and extends recent studies. Other groups have shown that HIV-1 CA-RNA and HIV-1 CA-DNA levels were maintained at higher levels in CSF cells than those in PBMCs, despite fully suppressive ART (7, 20). However, with the Double R assay, we were able to detect even higher levels of HIV-1 CA-RNA in 88% of CSF samples, as opposed to 9% in a previous report from a similar cohort of patients (7) and 76% for CA-DNA vs. 48% in one study previously (7) and 53% in another study (20). The differences in detection rates likely have two explanations. First, the very low numbers of CSF cells hamper the sensitivity of standard real-time PCR assay or even the Droplet Digital PCR assay. Second, the Double R assay is much more sensitive (9)–this is true even with adequate cell numbers as per our previous report in which we demonstrated high levels of HIV-1 CA-transcriptional activity in PBMCs despite virally suppressive ART (10). Our results are particularly supported by a recent report, using the single-cell RNA sequencing (scRNAseq) approach, that CD4+ T cells in CSF contain HIV RNA transcripts, in a very small number of cells (21). In that report, the small sample size did not allow an association with cognitive impairment (21), but future studies should be informative, although the use of oligonucleotide-labeled monoclonal antibodies to formally identify CD4+ T cells vs. other cell types will be required, as their transcripts of CD4 were not always detected by scRNAseq (21).

Importantly, our results demonstrate that there is a lot of transcription already, that the reservoir is active, such that further activation of the HIV promoter by “latency-reversing agents” could be redundant, consistent with many trials using the “Shock and Kill” approach, having little or no effect so far on HIV reservoirs (22).

The association between HIV-1 CA-RNA levels in CSF cells and concurrent brain injury is in keeping with the continued pathogenetic importance of HIV-1 in the CNS. Previously, it has been considered that brain injury in the context of viral suppression with ART (as assessed by the absence of cell-free HIV-1 in the CSF and plasma) was the consequence of pre-ART damage, intermittent low-level replication, comorbidities, and other non-HIV factors (4). Our findings of very high levels of HIV-1 RNA and DNA intracellularly make it likely that HIV-1 *still* plays a central role in neuropathogenesis. Some data have pointed to HIV-1 DNA per se in the CNS causing neuroinflammation and brain injury (23, 24). The stronger relationship of HIV-1 CA-RNA levels in CSF cells with brain injury, compared to DNA, in our study emphasizes the importance of transcription, though DNA alone may still play a role, albeit a lesser one. The nature of that transcription, however, requires further explanation. Our data relate to intracellular transcriptional activity in the context of ART–what is being measured is not necessarily cell-free whole virions (as measured by standard RNA assays), but rather RNA and probably viral components before assembly into whole virions. As such, the findings indicate the presence of HIV-1 components such as Tat, Nef, Vpr, and Env, some of which are neurotoxic and immunogenic (3, 11–15).

We defined brain injury in the current study in terms of abnormalities in ^1^H MRS of the brain and using a composite of brain major metabolites which captures the shared variance between them. This data reduction approach produces a statistically robust outcome (25), which was ideal in the context of our sample size where too many comparisons could have led to spurious findings. Using this composite brain score injury score, we further confirm a large pool of studies which have shown that ^1^H MRS is a robust marker of brain damage in treated and virally suppressed PLHIV whether they have HAND or not (1, 26–29). This body of research also shows that NAA and creatine reduction (axonal/neuronal damage; reduced bioenergetics) are abnormal in PLHIV despite successful ART with viral suppression. This is in line with our finding that NAA and creatine reduction showed a strong relationship with HIV-1 CA-RNA transcription in CSF cells.

The finding of a large relationship between HIV-1 CA-RNA in CSF and PBMCs is novel and suggests that CSF HIV-1 CA-RNA is chiefly being driven from the periphery by PBMCs. Plausibly, this could occur through CD4+ T cells trafficking through the CNS with the involvement of local CXCL10 (IP-10) production, as shown in the model in Fig. 4*B* (and see below). However, the HIV-1 CA-RNA and CA-DNA levels in the CSF are not just a reflection of the periphery, as the levels of HIV-1 RNA and DNA per CSF cell were significantly greater than those in PBMCs.

The CSF cellular origin of the HIV-1 RNA and DNA was probably the nongut homing CXCR3+ CD49d+ integrinß7-CCR5+ memory CD4+ T cells. These were the most populous CD4+ T cell type, while there were very few monocytes. Others have also shown the CSF cells in HIV-1-infected individuals to be composed mainly of CD4+ T cells and few monocytes (30). Farhadian et al, using single-cell RNA sequencing, also identified microglial type cells despite only accounting for <5% of CSF cells (31). Previous studies of PBMC have shown that circulating CXCR3+ CD4+ T cells contained the highest levels of integrated HIV DNA, relative to other subsets of memory CD4+ T cells (32), and also contained the highest levels of replication-competent HIV (33). Given that existing and published data confirm the dominance of CD4+ T cells in the CSF, that CD4+ T cells are the most permissive cell type for HIV-1 infection, and that CD4+ T cells carry the highest burden of HIV-1 in the blood, it is highly likely that these cells are the source of CA HIV-1 RNA transcripts in the CSF.

In contrast, it appears that in the cohort in the current study, it is very unlikely that their circulating monocytes are contributing substantially to the HIV-1 RNA transcripts in the CSF cells. In support of this, a very recent study of the minority of patients on ART with CSF viral “escape” found some evidence of the host T cell marker CD26 embedded in the envelope of cell-free HIV-1 in the CSF, consistent with a CD4+ T cell origin, rather than monocytes (5). Nevertheless, the possible roles of intermediate CD14+CD16+ monocytes, relatively rare in the circulation, but apparently enriched in the CSF cells, and microglia-type cells in the CSF (31), cannot be dismissed, but is still likely to be minor, based on the cell numbers. A recent report supports this conclusion, using scRNAseq, which found HIV RNA transcripts in cells imputed to be CD4+ memory T cells, by transcriptomic clustering, and not in microglial-type cells in the CSF (33). However, these latter results, with a very small sample size, need to be confirmed in future studies.

Several aspects of our data support HIV-1 compartmentalization in the CNS, similar to previous studies (34–36). The cellular load of HIV-1 was greater in the CSF compared to blood, the cellular expression of CCR5 was greater, and perhaps most interestingly the nongut homing CD4+ T cell phenotype was enriched in the CSF. The main chemokine ligand for CXCR3, namely CXCL10 (IP-10), has also been shown to be elevated in the CSF of untreated HIV+ patients (37, 38), correlating with CSF cell-free HIV-1 RNA (37–40), leukocyte numbers in CSF (37, 39), and HAND (39). Plasma levels of CXCL10 (IP-10) remain elevated compared to HIV-uninfected controls despite suppressive ART (41). Overall, our data strongly suggest that CXCL10 (IP-10) is likely to lead to directed trafficking of circulating infected CXCR3+ CD49d+ ß7-negative CD4+ T cells into the CNS.

Therefore, our findings can be integrated into a model of neuropathogenesis that extends previous models into the virally suppressive ART era. CD4+ T cells, as part of normal immune surveillance, enter the CNS. This model as shown in Fig. 4*B* is proposed to guide future studies. Some of the trafficking CD4+ T cells exhibit high levels of HIV-1 RNA transcripts and are infected with replication-competent HIV-1 despite virally suppressive ART, as we have previously shown (10), and can intermittently produce low amounts of HIV-1. This in turn leads to infection of perivascular macrophages, astrocytes, microglia, and pericytes (13, 42, 43). Even those CD4+ T cells that do not have replication-competent HIV-1 still harbor high levels of HIV-1 RNA that can be translated into viral components such as Tat, Env, Nef, and Vpr that are well described to be neurotoxic and immunogenic, (3, 11–15). Of these, Tat can be detected in exosomes in the CSF (17). A recent study showed that defective proviruses can produce transcripts with open reading frames that can be translated into Gag and Nef (44). Further, under virally suppressive ART, CNS cells are still likely to have HIV-1 RNA and DNA intracellularly, which can lead to injury. A previous study found that prematurely terminated short transcripts from HIV proviruses are readily detected in PBMCs from treated patients, and that they were associated with T cell activation (45). This model may explain why brain damage still occurs despite virally suppressive ART and why it is mild in the vast majority. ART significantly reduces the dissemination of replication-competent HIV-1 but is ineffective at inhibiting transcription, and translation of viral components, from HIV-1 integrated into the host DNA. Productive infection with whole virus formation is inhibited by ART, but not a restricted form of infection where potentially toxic viral components are still made. This model may also explain the therapeutic paradox of HAND–potent antiretroviral drugs are effective against the most severe form of HAND but not against the mildest/chronic form (i.e., the current sample), as the Achilles’ heel of ART is the lack of therapy targeting HIV-1 transcription.

Our study has several limitations. First, this is an observational study. However, it is increasingly accepted by statisticians (46, 47) that observational studies are powerful studies to detect a robust effect, especially when they are significant and with high effect size, when the samples are well characterized, when major outcomes are measured using standard and robust methodologies, and when the design and statistical approach control for key major factors, as in the current study. Second, the sample size was relatively small. Nevertheless, we demonstrated robust and at least medium to large effect sizes for HIV-1 RNA transcript levels on ^1^H MRS analyses. Third, the current sample had a restricted range of cognitive performance variability. Because of this homogeneity, it was not possible to determine any association between cognitive performance and the Double R assay biomarkers. The study is ongoing, and available longitudinal results suggest consistency of transcription results for CSF cells. Finally, the sample was composed only of men who were White Australians of English-speaking background. While this represents the most common demographic characteristics of the Australian HIV epidemic, further studies in more diverse samples will be important.

In conclusion, our study demonstrates high levels of HIV-1 CA-RNA transcriptional activity and DNA in CSF cells, which are associated with in vivo brain injury and which are most likely driven by trafficking memory CD4+ T cells. These results suggest that HIV-1 still has a central role in neuropathogenesis in the ART era. Indeed, our recent publication showing cell-associated HIV-1 RNA transcripts at much higher levels in memory CD4 T cells from most patients (10) than had previously been reported (48) is consistent with our current results and with hypotheses that HIV proviruses are not transcriptionally silent (49). Consequently, current ART needs to be extended to target inhibition of the HIV-1 promoter.

## Methods

### Participants.

The 16 study participants (Table 1) were HIV-1-infected males on fully suppressive ART (as assessed in both blood and CSF) who were enrolled into an ongoing prospective study of CNS HIV-1 latency and NeuroHIV biomarkers (ClinicalTrials.gov Identifier: NCT02989285). To be included in this current study, participants were required to have had stable HIV infection with viral suppression for at least 6 mo and sufficient fresh blood and CSF had to be available.

The study protocol was approved by the St. Vincent’s Hospital Human Research Ethics Committee (HREC/15/SVH/425), and all participants provided written informed consent prior to enrolment.

### Collection and Processing of CSF Cells and PBMCs.

CSF and anticoagulated peripheral blood were collected via lumbar puncture and phlebotomy, respectively, during one of the five study visits (a screening/baseline visit and four 6-monthly follow-up visits over 24 mo).

CSF samples (median 12.6 ml; IQR: 10.4–13.7 ml) were centrifuged at 400*g* for 15 min. CSF was carefully aspirated, and the cell pellets were resuspended in 1 ml Dulbecco’s Ca^2+^ and Mg^2+^-free PBS (Gibco, Life Technologies, Paisley, UK) containing 2% fetal calf serum (FCS; Gibco).

A 100 µl aliquot of CSF cell suspensions was analyzed by flow cytometry (see below), and the remaining 900 µl CSF cell suspensions were centrifuged at 5,000*g* for 3 min to obtain the CSF cell pellets, which were then subjected to extraction of total nucleic acid for HIV-1 molecular analysis by the Double R assay (described below).

Acid-citrate dextrose (ACD) anticoagulated blood samples were obtained on the same date as the lumbar puncture. PBMCs were prepared from the blood samples by standard density gradient centrifugation on Ficoll-Hypaque Plus (GE Healthcare, Chicago, IL, USA). PBMCs were cryopreserved in heat-inactivated, filter-sterilized, FCS (Gibco) containing 10% dimethyl sulphoxide (DMSO; Sigma Aldrich, MO, USA) using a controlled rate freezer (Planer, Middlesex, UK) and stored in vapor-phase liquid nitrogen.

### The Double R Assay on the πCode Endpoint PCR Platform.

We used our recently described assay to detect HIV-1 CA-RNA transcription and total HIV-1 DNA copies (9, 10). Briefly, the primers and probes used in this assay target the highly conserved “R” region in both the 5′- and 3′- “LTR” regions, which permits detection of total spliced and unspliced mRNA transcripts, using reverse transcription, as well as, in parallel, integrated HIV-1 proviral DNA without RT (9, 10). The amplicons are then read out with the highly sensitive image analysis of precision image pi-code (πCode) MicroDiscs, as previously described (9, 10). The overall method results in at least 27-fold higher sensitivity than that of the current real-time PCR assays (9, 10).

For PBMCs, HIV-1 CA-DNA and RNA were extracted using the Maxwell RSC automated extraction platform (Promega), with the Maxwell RSC Buffy Coat DNA kit (Cat No. AS1540, Promega) and Maxwell RSC Simply RNA Tissue kit (Cat No. AS1340, Promega), respectively, according to the manufacturer’s protocol. The RNA assay for PBMCs uses the PrimeScript One-step RT-PCR kit (Takara Bio, Kusatsu, Shiga, Japan). The Double R assay can detect as few as two OM10.1 cells (containing single integrated HIV-1 provirus per cell) when diluted in as many as 10^6^ uninfected cells (9) (see *SI Appendix*, *Method* for detailed analysis and *SI Appendix*, Tables S5–S8). Quantification of HIV-1 copy number per patient sample was determined using set-4 and set-6 probes (9, 10) with a standard curve generated with HIV-1 plasmid controls: 0.73, 2.2, 6.6, 20, 59, 177, 533, and 1,600 HIV-1 copies /μl. The HIV-1 copy number for each sample, from the average value of duplicate reactions, was normalized with the CD4+ T cell count number, which was obtained by the flow cytometry analysis (below). HIV-1 copy number per one million CD4+ T cells was used as the standardized unit.

CSF cell nucleic acids were obtained using total nucleic acid extraction (Maxwell RSC viral Total Nucleic Acid Purification kit (Cat No. AS1330, Promega, Madison, WI, USA). Total HIV-1 RNA and HIV-1 DNA copy numbers were measured using the PrimeScript One-step RT-PCR kit, as above for PBMC. In parallel, HIV-1 DNA copy numbers were identified using the same detection procedure of the PrimeScript One-step RT-PCR kit (Takara) without the addition of RT enzyme. HIV-1 RNA copy numbers were therefore calculated by subtracting the HIV-1 DNA copy number from the total HIV-1 copy number (see *SI Appendix*, *Method* for detailed validation of this analysis and *SI Appendix*, Table S8).

For PBMCs, HIV-1 CA-DNA and RNA were extracted using the Maxwell RSC automated extraction platform (Promega), with the Maxwell RSC Buffy Coat DNA kit (Cat No. AS1540, Promega) and Maxwell RSC Simply RNA Tissue kit (Cat No. AS1340, Promega), respectively, according to the manufacturer’s protocol.

From PBMCs, purified CD14+ monocyte and CD14-negative preparations (see below) were divided into two portions each with equal numbers of cells, followed by centrifugation at 5000*g* for 5 min. One cell pellet was used for DNA extraction and the other cell pellet was used for RNA extraction (as for unfractionated PBMC, above).

### Detection of Cell-Free HIV-1 in CSF.

CSF cell-free virus was measured using the standard Roche Amplicor assay with a lower limit of detection of <80 copies/ml. HIV-1 RNA levels were also quantified in 7 ml CSF using the single-copy HIV-1 RNA assay (SCA). All samples quantified by SCA were centrifuged to remove cellular debris and run in triplicate, with a lower limit of detection of 0.3 copies/ml, as previously described (10).

### CD14+ Monocyte Isolations from PBMCs.

CD14+ monocytes were isolated from cryopreserved PBMCs, which had been thawed and resuspended in PBS with 2% FCS, using positive selection on the automated RoboSep platform (CD14+ cell-positive separation kit, Cat No. 17858RF, StemCell Technology, Vancouver, Canada), according to the manufacturer’s directions. The number of CD4+ T cells in the isolated CD14+ monocyte populations and in the CD14-negative fraction was accurately counted using TruCount tubes (BD Biosciences). CD14+ monocyte fractions were routinely highly purified and only contained median 0.26% (IQR: 0.19–0.35) contaminating CD4 T cells.

### Flow Cytometry Analysis.

As described above, CSF samples were centrifuged at 400*g* for 15 min. CSF was carefully aspirated and the cell pellet resuspended in 1 ml Dulbecco’s Ca^2+^ and Mg^2+^-free PBS containing 2% FCS. A 40 µl aliquot of these CSF cells was used to accurately count CD4+ T cells and other PBMC types, using a 12-color monoclonal antibody panel (*SI Appendix*, Table S1 section (a)), in TruCount tubes (BD Biosciences, San Jose, CA, USA), according to the manufacturer’s directions, on a 5-laser Fortessa flow cytometer (BD Biosciences), as previously described (50). A 60 µl aliquot was separately further analyzed for memory T cell subsets using an 18-color monoclonal antibody panel (*SI Appendix*, Table S1 section (b)), on the Fortessa flow cytometer, as previously described (51). Cryopreserved PBMC samples were thawed and analyzed for memory T cell subsets using the same 18-color monoclonal antibody panel (*SI Appendix*, Table S1 section (b)).

### ^1^H Magnetic Resonance Spectroscopy.

^1^H MRS brain scans were conducted within 4 wk of CSF and PBMC collection for the majority (60%) of participants (median: 0 wk). The ^1^H-MRS protocol used in this study has been described previously (52). Briefly, spectra were acquired on a Phillips 3 T Ingenia scanner (Philips, Best, Netherlands) using a 32-channel head coil. Cerebral metabolite concentrations were quantified using point-resolved spectroscopy (PRESS: point-resolved spectroscopy; TE = 40 ms; TR = 2,000 ms) in the FWM, PCC, and caudate nucleus. jMRUI v3.0 (53) with Advanced Method for Accurate, Robust and Efficient Spectral (AMARES) algorithm (54) was used to analyze fitted spectra, which included NAA, choline, creatine (Cr), *myo*-inositol (mIo), and glutamate (Glu). The spectra were expressed as ratios in relation to unsuppressed water signal (H_2_O). Spectra values were normalized using a reference sample of demographically comparable 54 HIV controls from our HIV-1 and Ageing Research Program (55) and converted to age-corrected z-scores.

Age-corrected spectra z-scores were highly correlated within each voxel (FWM: ρs = 0.20–0.84; PCC: ρs = 0.56–0.89; caudate ρs = −0.02–0.66). The metabolites’ data by voxel were amenable to data reduction using principal component analysis and maximum likelihood method, a method of choice to reduce ^1^H MRS data (25). We extracted a single in vivo composite score for each voxel accounting for 67% of common variance in the FWM, 84% in the PCC, and 71% in the caudate spectra (see *SI Appendix*, Fig. S3 for relative contributions of each metabolite to voxel composite scores). Lower ^1^H MRS composite scores reflected higher levels of in vivo brain injury.

### Neuropsychological Methods.

We used a standard neuropsychological battery (*SI Appendix*, *Method*) covering seven cognitive domains, in line with the Frascati recommendations (56) and NeuroHIV research internationally (57), that has previously been demonstrated by our group to be sensitive to HIV-associated neurocognitive disorder (HAND) (58, 59).

### Statistical Analysis.

Standard curves from known concentrations of HIV-1 plasmid copy numbers were generated with GraphPad Prism v7 (GraphPad Software). HIV-1 CA-RNA transcription and HIV-1 CA-DNA copy numbers (as measured by the Double R assay) in CSF cells vs. PBMCs were compared by nonparametric Mann–Whitney *U* tests. Differences in the proportions of subsets of memory CD4+ T cell subsets, between paired CSF cell and PBMC samples, from individual participants, were compared using paired Wilcoxon nonparametric tests. Pearson’s correlations were used to analyze the association between HIV-1 CA-RNA and HIV-1 CA-DNA levels within each cell type.

ART initiation time and history of ART exposure often vary in people with long-term HIV infection. Therefore, to account for the initiation timing of suppressive ART on HIV-1 CA-DNA and RNA, we divided the total duration of HIV-1 infection into two time-related variables that were examined separately: time from seroconversion to suppressive ART initiation and duration of suppressive ART exposure. Nonparametric Spearman’s rank correlations were used to analyze the relationships of HIV-1 CA-RNA and HIV-1 CA-DNA levels in CSF and PBMCs with suppressive ART exposure. We additionally classified participants as being either “early” or “late treated,” depending on whether or not they commenced suppressive ART within vs. more than 12 mo postseroconversion. The CNS Penetration Efficiency (CPE) rank score is provided in Table 1 and was used to further assess whether there was any association between the study outcomes and variations in ART regimen types.

To characterize the association between HIV-1 CA-RNA transcription and HIV-1 CA-DNA copies with ^1^H MRS-related brain injury, we conducted a series of hierarchical linear regression models. Each model contained one of the four Double R assay biomarkers (i.e., CSF CD4+ HIV-1 CA-RNA transcription, CSF CD4+ HIV-1 CA-DNA, PBMC CD4+ HIV-1 CA-RNA transcription, or PBMC CD4+ HIV-1 CA-DNA) as the criterion variable. In the first step, one of the three ^1^H MRS voxel composite scores was entered. In the second step, early vs. late suppressive ART initiation was entered as a covariate. We also considered models where time to suppressive ART initiation was entered instead as a continuous covariate at step 2. However, we retained the models containing the dichotomous suppressive ART exposure variable as it provided a similar or slightly improved fit each time. Parameter estimates and standardized β effect sizes were extracted at each step of model fitting and compared.

For two participants, two sets of PBMC/CSF cell pairs were collected 6 mo apart. We used these additional samples to assess the longitudinal stability of the molecular-based analyses.

## Supplementary Material

Appendix 01 (PDF)

## Data Availability

All study data are included in the article and/or *SI Appendix*.
