# Supplementary material for: Elevation of cell-associated HIV-1 transcripts in CSF CD4+ T cells, despite effective antiretroviral therapy, is linked to brain injury
Source: Proc Natl Acad Sci U S A. 2022 Nov 21;119(48):e2210584119. doi: 10.1073/pnas.2210584119 (PMC9860316; doi:10.1073/pnas.2210584119)
Supplement: Supplementary file 1 — Appendix 01 (PDF) [file pnas.2210584119.sapp.pdf]

PNAS

Supporting Information for

Elevation of cell-associated HIV-1 transcripts in CSF CD4+ T cells, despite effective antiretroviral therapy, is linked to brain injury

Kazuo Suzuki[1,2*], John Zaunders [1,2*], Thomas M. Gates[3,4,5,6], Angelique Levert[1], Shannen Butterly[1], Zhixin Liu[7], Takaomi Ishida[8], Sarah Palmer[9], Caroline D. Rae[10,11], Lauriane Jugé[10,11], Lucette A. Cysique[3,4,5,6], Bruce J. Brew [2,3,4,5,11,12*].

* These authors contributed equally

Email:

k.suzuki@amr.org.au

j.zaunders@amr.org.au

Bruce.Brew@svha.org.au

This PDF file includes:

Supporting text
Tables S1 to S8
Figures S1 to S3
SI References

Supporting Information of Neuropsychological Methods and Results

Methods

The standard neuropsychological battery (provided below) covered 7 cognitive domains, in accordance with the Frascati recommendations (1) and NeuroHIV research internationally (2), that has previously been demonstrated by our group to be sensitive to HAND (3, 4). The test battery was administered by a qualified neuropsychologist or clinical psychologist and typical completion time was around 2-3h. The test battery comprised a combination of standardized pencil-and-paper neuropsychological tests supplemented with the brief computerized battery, CogState™ (5). Additionally, pre-morbid intellectual functioning was estimated using the Wechsler Test of Adult Reading (WTAR) (6) or a demographics-based regression equation when the WTAR was not administered (http://www.assessmentpsychology.com/iq-estimates-3.htm).

Psychological measures included the Depression, Anxiety, and Stress Scales (DASS-21) (7) and Mini International Psychiatric Interview (MINI v5.0.) (8). Medication adherence was assessed using the Simplified Medication Adherence Questionnaire (SMAQ) (9). Functional status was measured using a modified version of the Lawton and Brody instrumental activities of daily living (IADL) questionnaire (10).

Overall neurocognitive impairment and the presence of HAND was determined using the Global Deficit Score (GDS) method (11), a battery-wide summary of impairment. We used the standard cut-off of GDS≥0.5 to define impairment which is a well-established marker of HIV-related brain injury. The GDS was computed as follows: raw scores on each measure were converted to normally distributed and demographically-uncorrected scaled scores, and then to demographically-corrected T-scores using one of two sets of regression-based, demographically-corrected norms deemed most sensitive for detecting mild levels of cognitive impairment given the individual participant's age, educational attainment and estimated pre-morbid full-scale IQ (FSIQ), as follows:

1) <u>Age<67 years and either WTAR-estimated FSIQ≥110 or ≥15 years of education</u> = Australian high-functioning male norms (Gates et al., unpublished observations). These norms were derived from a high-functioning HIV-uninfected (HIV-) MSM control sample (n=70) who participated in our NeuroHIV research program at the University of New South Wales and St Vincent's Hospital, Sydney, Australia. The norms provided corrections for age and

education in high-functioning individuals (the gender and ethnicity composition of the normative sample were homogenous, similar to the current sample. Therefore, corrections were not made for these demographic factors).

2) a) Age≥67 years; or b) Age<67 years, WTAR-estimated FSIQ<110, and <15 years of education = demographically-corrected US norms (see Supplementary Table 5).

Demographically-corrected Tscores on each measure were then converted into deficit scores (ranging from 0-5), which were in turn averaged to generate the GDS. A GDS equal or greater than 0.5 was used to classify impairment. IADL decline in at least two activities of daily living discriminated between Asymptomatic Neurocognitive Impairment (ANI) and Mild Neurocognitive Disorder (MND). There were not cases with HIV-associated dementia (i.e., severe cognitive impairment and severe IADL decline).

Results

We found that the sample had a relatively restricted range of cognitive performance variability and while the cases with HAND has lower performance (as expected, Mean T-scores = 45.5) than the NP-normal cases (mean T-scores = 49.5), this was just significant at p=.04. Four of the non-HAND participants had borderline performance; while non the HAND cases were severely impaired (i.e., ANI or MND). In addition, our capacity to detect a relationship with HIV-1 CA RNA transcripts and DNA was affected by the fact that in half of the HAND cases the transcripts were not detected. We therefore used the Mean-T score (rather than the HAND status) to assess the association with the transcripts and none were significant.

The lack of power to correlate HAND classification with the virological and immunological parameters and the restricted range of performance in the sample represent limitations of the current study. However, in people with long-term HIV infection, cross-sectional analysis of cognitive impairment may be difficult to interpret, especially if there is good compensation as it is probably the case in mild HAND cases, obscuring any relationship with the underlying viral dynamics. In contrast, the ^{1}H MRS is closer to the actual level of brain injury compared to what patients can achieve neuropsychologically. The study is ongoing and available longitudinal results suggest consistency of transcription results for CSF cells. Cognitive decline may become apparent with further longitudinal neuropsychological testing.

Neuropsychological Test Battery

Cognitive Domain	Measure	Australian Norms (Reference)	US Norms (Reference)
Attention/working memory	CogState One-Back Task – Accuracy	(12) *	(12) *
Speed of information processing	Trail Making Test – Part A	Gates et al. (unpublished observations)	(13)
	WAIS-III Digit-Symbol Coding	Gates et al. (unpublished observations)	(14)
	CogState Detection Task – Speed	(12) *	(12) *
	CogState Identification Task – Speed	(12) *	(12) *
	CogState One-Back Task – Speed	(12) *	(12) *
Motor coordination	Grooved Pegboard – Dominant Hand	Gates et al. (unpublished observations)	(13)
	Grooved Pegboard – Non-Dominant Hand	Gates et al. (unpublished observations)	
New learning	Hopkins Verbal Learning Test – Revised – Total Learning	Gates et al. (unpublished observations)	(15) †
	CogState One Card Learning Task – Accuracy	(12) *	(12) *
Memory	Hopkins Verbal Learning Test – Revised – Delayed Recall	Gates et al. (unpublished observations)	(15) †
Language	Letter Fluency – FAS	Gates et al. (unpublished observations)	(13)
	Animal Fluency	Gates et al. (unpublished observations)	(13)
Executive functioning	Trail Making Test – Part B	Gates et al. (unpublished observations)	(13)
	Wisconsin Card Sorting Test – 64 Card Version (Computerized) – Perseverative Errors	(16) ‡	(16)

* CogState norms are based on an Australian sample and corrected for age and gender only. Equivalent US norms are not available; Australian norms were therefore used for all participants.

† US norms from the HVLT-R Professional Manual (15) are adjusted for age only. However, they were used in preference to other available demographically-corrected US norms (e.g., Norman et al. (2011) (17)) as the latter were shown to significantly underestimate performance in the Australian HIV- control sample from which the local norms were derived, whereas performance remained within expectations on the HVLT-R Professional Manual (15) US norms.

‡ WCST-64 was not completed by the Australian HIV- control sample from which the local norms were derived; US norms were therefore used for all participants.

Supporting Information of the Double R assay on the πCode End-Point PCR platform (OM10.1 detection)

In order to define the assay detection sensitivity (see Supplementary Table 6), we used OM10.1 cells (containing single integrated provirus, ARP-1319, Human T-Cell Lymphoma, AIDS reagent program NIH). We made five-fold serial dilution series in the presence of one million uninfected HUT-78 cells (ARP-89, Human T-Cell Lymphoma, AIDS reagent program NIH).

No.1 setting containing 1000 OM10.1 cells in 1 x 10^6 HUT-78 cells
No.2 setting containing 200 OM10.1 cells in 1 x 10^6 HUT-78 cells
No.3 setting containing 40 OM10.1 cells in 1 x 10^6 HUT-78 cells
No.4 setting containing 8 OM10.1 cells in 1 x 10^6 HUT-78 cells
No.5 setting containing 1.6 OM10.1 cells in 1 x 10^6 HUT-78 cells
No.6 setting containing no OM10.1 cell in 1 x 10^6 HUT-78 cells

Two sets of identical 6-tube serial dilutions of OM10.1 and HUT-78 mixtures sample in 1ml, as above, were prepared in Eppendorf tubes, followed by centrifugation at 6000g for 5 min to form cell pellets. After removing supernatant, one set of the 6 tubes was used for CA HIV-1 RNA transcripts analysis. The other set was used for CA HIV-1 DNA analysis.

1). CA HIV-1 RNA transcripts analysis

RNA was extracted using the Maxwell RSC automated extraction platform (Promega) using Maxwell RSC Simply RNA Tissue kit (Cat No. AS1340, Promega) with 200μl of elution buffer. We used PrimeScript One-Step RT-PCR kit (Takara Bio, Kusatsu, Shiga, Japan), as described previously (18, 19). We adapted previously published well-described methodology for Real-Time DNA PCR for mRNA quantification (20, 21).

A DNA standard can be synthesized by cloning the target sequence into a plasmid or produced as a purified PCR product, for use in a two-step real-time PCR, with reverse transcription of sample RNA as the first step (as reviewed in (20)). DNA standards have been shown to have a larger quantification range and greater sensitivity, reproducibility, and stability than RNA standards. In contrast, the SP6/T7 in-vitro transcription method can be used to generate single strand RNA from plasmid DNA sequences cloned downstream of the

SP6 or T7 promoter to generate RNA standards, but these RNA standards are not as stable as the corresponding DNA standards (20). We applied this approach for a one step Reverse Transcriptase PCR analysis (reviewed in (21)).

We did the following five critical analyses to validate the DNA standard for one-Step Reverse Transcriptase PCR analysis

1. Analysis of DNA co-extraction levels in RNA extracted solution obtained by Maxwell RSC Simply RNA Tissue kit
2. RNA integrity analysis
3. No presence of copurification of inhibitors in the RT reaction step
4. No presence of nuclease for extended storage.
5. Identification of the assay limitation factor in the RT reaction step in one step PCR reaction

1). Absence of any DNA co-extracted with RNA using the Maxwell RSC Simply RNA Tissue kit (see Supplementary Table 7)

Identification of DNA co-extraction levels in RNA extracted solution was essential. We carried out both analyses of PrimeScript One step RT-PCR with and without RT reaction step (Supplementary Table 7). The data indicated that DNA co-extraction levels in RNA solution were equal to zero, indicating the DNase treatment step during an automated extraction in Maxwell RSC machine succeeded in completion of removal of any DNA. HIV-1 quantification with the One-Step RT-PCR was totally dependent on the newly synthesized cDNA, generated in the initial Reverse-Transcriptase (RT) reaction at 50°C for 2min, from the positive strand of HIV-1 mRNA with the specific PCR primer. The next step DNA PCR amplification cycles (which followed the initial RT reaction in the same tube) detected only the newly synthesized cDNA from the initial RT reaction step. The quantified HIV-1 RNA data, generated by the one-step RT-PCR had no contribution of co-extracted HIV-1 DNA levels in the RNA extracted solution.

2). RNA integrity analysis

We assessed RNA integrity using the TapeStation analysis system (Agilent, Santa Clara, CA, USA). We confirmed that The RNA Integrity (RIN) Number was above RIN of 8 for

extracted RNA using Maxwell RSC Simply RNA Tissue kit to identify generation of the best ratio of 28S and 18S rRNA peaks.

3). No presence of copurification of inhibitors in the RT reaction step

There are a large number of components within whole blood samples that may inhibit the RT reaction step, as well as the following DNA PCR (20, 21) for example the heme compound. We routinely use isolated PBMC, free of red blood cells, so that heme compound is not present. Nevertheless, we assessed inhibitor effect together with the RT reaction step analysis (as described below in section #5).

4). No presence of nuclease for extended storage.

Analysis of RNA stability is important to exclude the presence of nuclease in the extracted RNA. We assessed any possible storage effect in the RIN numbers. We found that the RIN number was maintained above RIN of 9 for extracted RNA using Maxwell RSC Simply RNA Tissue kit for 10-day storage at 4°C, indicating no presence of nuclease in the RNA extracted solution using the Maxwell RSC Simply RNA Tissue kit.

5). Identification of the assay limitation factor in the RT reaction step in one step PCR reaction (see Supplementary Table 8)

We found that the assay is strictly dependent on the amount of target RNA in the samples. We identified the amount of the newly synthesized cDNA levels didn't change with extended reaction time of the initial RT reaction at 50°C: 1, 2, 4, 6, 10 minutes (see Supplementary Table 8). The data indicated that there was no difference in generated MFI (Mean Fluorescent Intensity) by the πCode End-Point PCR with extended RT reaction time. This data also suggested that there was no copurification of inhibitors in the RT reaction step.

The same data also indicates that the necessary excess amount of the specific PCR primer was present in the One-Step RT reaction. Therefore, duration of the initial RT reaction and amount of the reverse primer are not assay limitation factors in the πCode End-Point PCR. Therefore, we used 2 minutes RT reaction at 50°C, which was long enough to convert of all positive strand of HIV-1 mRNA present in the sample to quantify CA HIV-1 RNA transcripts.

Overall, the Supplementary Table 6 identified that we were able to detect CA HIV-1 RNA transcripts in less than 2 OM10.1 cells in the presence of one million of uninfected HUT-78

cells. The quantified HIV-1 RNA in five-fold serial dilution showed close to 5-fold differences in HIV-1 copy numbers in the dilution series, indicating that the πCode End-Point PCR was optimized to linearly quantify HIV-1 RNA in our developed assay system.

1). CA HIV-1 DNA analysis

DNA was extracted using the Maxwell RSC automated extraction platform (Promega) using Maxwell RSC Buffy Coat DNA kit (Cat No. AS1540, Promega) with 250µl of elution buffer. We used PrimeScript One step RT-PCR kit (Takara Bio, Kusatsu, Shiga, Japan) without addition of RT enzyme as described previously (18, 19). We used the HIV-1 plasmid controls: 0.73, 2.2, 6.6, 20, 59, 177, 533 and 1,600 HIV-1 copies /µl. PCR cycle conditions were identical as for our One-Step RT PCR, except omission of the initial RT reaction step. Supplementary Table 6 shows that we were able to detect CA HIV-1 DNA in 8 OM10.1 cells in the presence of one million uninfected HUT-78 cells. The quantified HIV-1 DNA in five-fold serial dilution tubes showed nearly 5-fold difference in HIV-1 copy numbers, indicating that the πCode End-Point PCR was optimized to quantify HIV-1 DNA in our system. The data in Supplementary Table 6 also indicated that OM10.1 showed very low corresponding levels of HIV-1 transcripts. The difference in copy numbers between HIV-1 DNA and CA HIV-1 RNA transcripts identified that HIV-1 RNA transcripts showed between 2-3 times higher copy numbers in HIV-1 RNA in each experimental setting of the serial dilution tubes, indicating minor ongoing transcription present in the HIV latent cells of OM10.1.

CSF cell analysis with the Double R assay on the πCode End-Point PCR platform (see Supplementary Table 9)

To identify HIV-1 CA HIV-1 RNA transcripts and HIV-1 DNA levels in CSF cells, we investigated methodology for small numbers of $CD4^{+}$T cells analysis. We used the same concept as in previously described the Dried Blood Spot analyses (DBS) based on the TaqMan probe Real-Time One-Step PCR assay (below). The DBS was used as an alternative procedure instead of standard whole blood to detect HIV-1 in resource limited countries. To achieve highest detection sensitivity in DBS analysis, Total Nucleic Acid (TNA) isolation was used to recover both CA-RNA/plasma HIV-1 RNA as well as CA-DNA, where those three components of HIV-1 are present in DBS (Suzuki et al, manuscript in preparation).

Furthermore, to achieve the highest sensitivity to detect HIV-1, the One-Step RT-PCR amplification was used to detect HIV-1 RNA as well as HIV-1 DNA in three automated analysis systems: Abbott M 2000, Roche Cobas Ampliprep/Cobas TaqMan, and Cepheid GeneXpert (22-25).

Although our CSF samples in this study did not contain any detectable HIV-1 RNA levels in the fluid phase, unlike in DBS samples, our CSF samples contained both CA-HIV-1 RNA and CA-HIV-1 DNA within the CSF cells. Therefore, we applied the same HIV-1 analysis approach used in DBS to adapt to CSF cell analysis. We did two critical assessments: i). selection of extraction method and ii). One-Step RT-PCR analysis for both HIV-1 RNA and DNA, to confirm the reported previous DBS analyses (22-25). We used two patients' PBMCs, prepared using the standard Ficoll-Hypaque Plus procedure. After determining $CD4^{+}$T cells by flow cytometry, we made three identical sets of four-fold serial dilution series in 1ml of PBMCs with Dulbecco's Ca^{2+} and Mg^{2+} free PBS containing 2% fetal calf serum. These diluted 1ml of PBMCs in Eppendorf tubes were centrifugated at 6000g for 5 min to form cell pellets. After removing supernatant, the three identical sets were used for three Maxwell extraction methods: DNA extraction (Cat No. AS1540), RNA extraction (Cat No. AS1340), and TNA extraction (Cat No. AS1330). The accurately counted $CD4^{+}$T cells are shown in the second column of Supplementary Table 9. The numbers of recovered CD4+ T cells from CSF samples (median: 3,605 cells), as shown in Figure 1a, therefore, the last two rows of Supplementary Table 9 are in the same range as the CSF cells, in this experiment.

The third column in the Supplementary Table 9 showed HIV-1 copy numbers by the One-Step RT PCR data by the πCode End-Point PCR with RNA extraction method. This RNA data is used as the reference in this evaluation, since RNA extraction solution with Maxwell system has no HIV-1 DNA contribution in the CA-HIV-1 RNA analysis (see above). The fourth column showed HIV-1 copy numbers by the One-Step RT PCR data with TNA extraction method. This TNA data in column four measures both CA-HIV-1 RNA and CA-HIV-1 DNA in the TNA extracted solution. The fifth column shows HIV-1 copy numbers by the DNA PCR analysis data with TNA extraction method, where we used the same PCR amplified condition as in One-Step RT RNA analysis, except without addition of RT enzyme. Therefore, the fifth column detects only HIV-1 DNA, while the sixth column shows the HIV-1 RNA copy numbers in the sample by subtraction of DNA copy number (column 5) from

the total TNA HIV-1 copy number (column 4). This data shows that the calculated number of HIV-1 RNA with the TNA extraction method (column 6) showed higher levels, compared with RNA only extraction method (column 3). It is noted that especially the last two rows, comparable to CSF cell counts, showed higher levels of isolated CA-HIV-1 RNA using the TNA method. Therefore, we used 2 aliquots from the TNA extraction method with One-Step RT PCR, with and without RT respectively, and analysis by the πCode End-Point PCR, to separately determine HIV-1 CA-RNA and CA-DNA levels in our CSF cell samples.

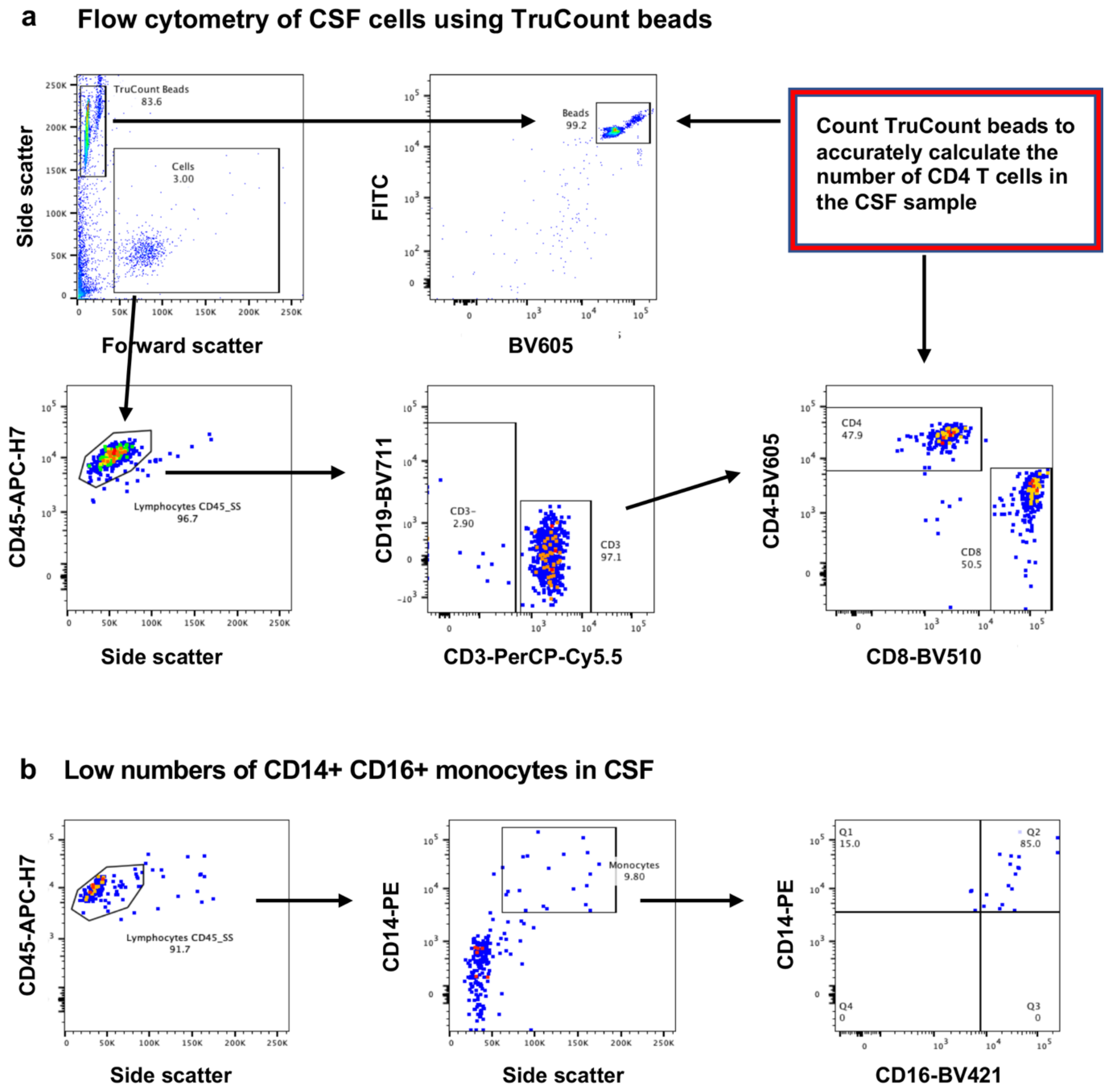

Fig. S1. (a) Representative flow plots showing the gating strategy to count TruCount beads and CD45+ cell types in CSF cell pellets, showing CD4+ and CD8+ T lymphocytes; and (b) the gating strategy to count CD14+ monocytes, and the presence of CD14+CD16+ intermediate monocytes in the same TruCount tubes.

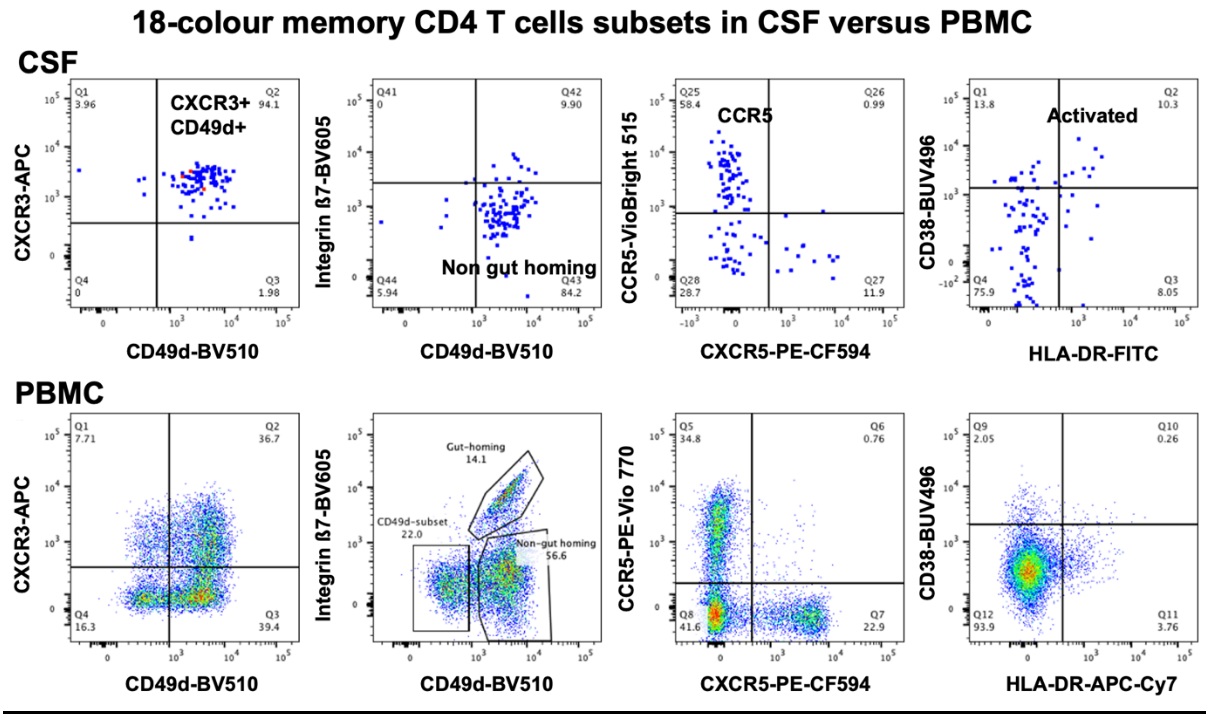

Fig. S2. Representative flow plots showing gated memory CD4 T cells from CSF cells (upper flow plots) and PBMC (lower flow plots), respectively, stained for, from left to right, CXCR3 vs CD49d; integrin ß7 vs CD49d; CCR5 vs CXCR5; and CD38 vs HLA-DR.

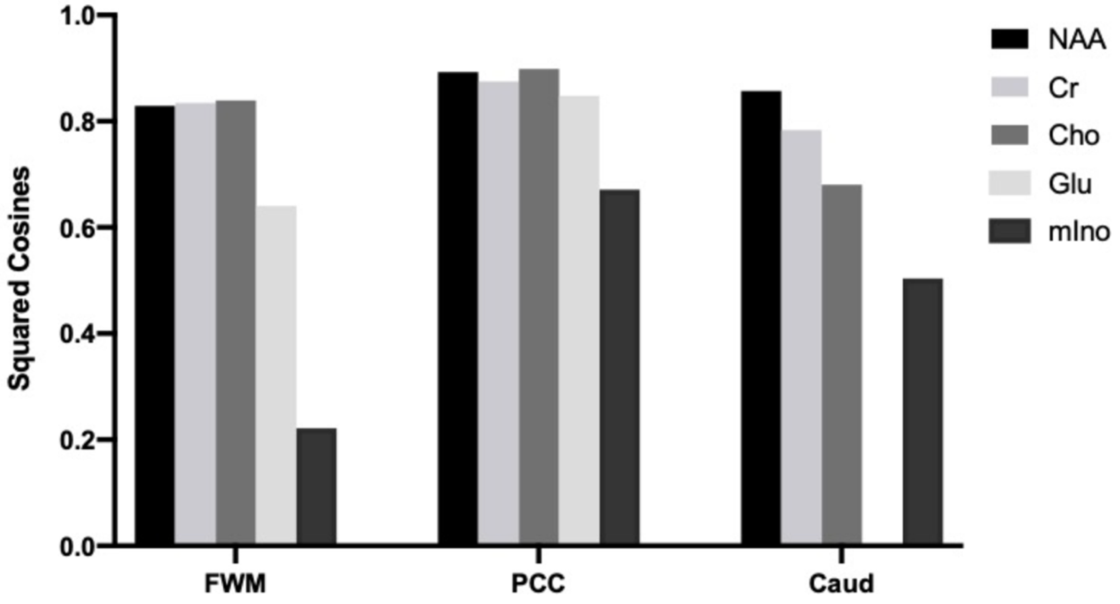

Fig. S3. Squared cosines of each metabolite with respect to each ^{1}H MRS voxel composite score. High relative contributions of multiple metabolites reflect strong intercorrelation permitting PCA. Note the caudate composite score consists of only 4 spectra; glutamate was not able to be quantified reliably.

Table S1. Monoclonal antibodies used in this study

mAb/ reagent	Clone	Fluoro-chrome	Supplier
**(a) TruCount tube**			BD
CD45	2D1	APC-H7	BD
CD56	NCAM16.2	APC	BD
CD27	M-T271	APC-R700	BD
HLA-DR	L243	FITC	BD
CD3	SK7	PerCP-Cy5.5	BD
CD16	3G8	BV421	BD
CD8	SK1	BV510	BD
CD4	RPA-T4	BV605	BD
CD19	SJ25C1	BV711	BD
CD123	7G3	BV786	BD
CD14	MøP9	PE	BD
CD38	HB7	PE-Cy7	BD
**(b) T cell phenotyping**			
CD28	CD28.2	APC	BD
CD27	M-T271	APC-R700	BD
CD62L	DREG-56	APC-eFluor780	eBioscience
CXCR3	REA232	Biotin	Miltenyi Biotec
Anti-Biotin	REA746	VioBright 515	Miltenyi Biotec
CD3	SK7	PerCP-Cy5.5	BD
CCR6	G034E3	BV421	BioLegend
CD49d	9F10	BV510	BioLegend
Integrin ß7	FIB04	BV605	BD
HLA-DR	G46-6	BV711	BD
CD127	HIL-7R-M21	BV786	BD
CCR5	REA245	PE	Miltenyi Biotec
CXCR5	J252D4	PE-CF594	BioLegend
CD25	M-A251	PE-Cy5	BD
CD161	REA631	PE-Vio770	Miltenyi Biotec
CD4	RPA-T4	BUV395	BD
CD38	HIT2	BUV496	BD
CD45RA	HI100	BUV 737	BD
CD8a	SK1	BUV805	BD

Table S2. ^{1}H MRS voxel composite hierarchical linear regression models

	HIV-1 RNA / CSF CD4 (Log_{10})				HIV-1 DNA / CSF CD4 (Log_{10})				HIV-1 RNA / 10^6 PBMC CD4 (Log_{10})				HIV-1 DNA / 10^6 PBMC CD4 (Log_{10})			
Predictors	$R^2/\Delta R^2$	B (SE)	β	p	$R^2/\Delta R^2$	B (SE)	β	p	$R^2/\Delta R^2$	B (SE)	β	p	$R^2/\Delta R^2$	B (SE)	β	p
Step 1	0.53				0.05				0.16				0.10			
FWM Composite		-0.26 (0.08)	-0.73	0.007		-0.08 (0.11)	-0.23	0.47		-0.14 (0.09)	-0.41	0.15		-0.12 (0.11)	-0.31	0.28
Step 2	0.07				0.21				0.37				0.37			
FWM Composite		-0.23 (0.08)	-0.67	0.01		-0.04 (0.10)	-0.12	0.69		-0.17 (0.07)	-0.47	0.04		-0.15 (0.09)	-0.38	0.12
Early/Late Treated		0.13 (0.11)	0.27	0.25		0.24 (0.15)	0.47	0.14		0.41 (0.14)	0.61	0.01		0.46 (0.17)	0.61	0.02
Step 1	0.38				0.09				0.12				0.08			
PCC Composite		-0.14 (0.06)	-0.61	0.03		-0.08 (0.08)	-0.30	0.34		-0.12 (0.09)	-0.35	0.21		-0.11 (0.10)	-0.28	0.31
Step 2	0.07				0.19				0.26				0.29			
PCC Composite		-0.13 (0.06)	-0.53	0.06		-0.05 (0.08)	-0.19	0.54		-0.08 (0.08)	-0.23	0.34		-0.06 (0.09)	-0.16	0.50
Early/Late Treated		0.14 (0.12)	0.28	0.29		0.23 (0.15)	0.45	0.16		0.34 (0.15)	0.52	0.05		0.41 (0.17)	0.55	0.04
Step 1	0.14				0.02				0.02				0.03			
Caudate Composite		-0.14 (0.10)	-0.38	0.21		-0.05 (0.11)	-0.15	0.65		-0.08 (0.15)	-0.14	0.62		-0.10 (0.17)	-0.17	0.55
Step 2	0.34				0.34				0.41				0.44			
Caudate Composite		-0.21 (0.09)	-0.57	0.04		-0.13 (0.10)	-0.35	0.24		-0.18 (0.12)	-0.34	0.06		-0.23 (0.13)	-0.37	0.12
Early/Late Treated		0.30 (0.12)	0.62	0.02		0.31 (0.14)	0.62	0.06		0.44 (0.15)	0.67	0.01		0.51 (0.16)	0.69	0.05

Participants were classified as being either “early” or “late treated”, depending on whether or not they commenced suppressive ART within vs more than 12 months post-seroconversion

Table S3. ^1H MRS NAA/H_2O hierarchical linear regression models

	HIV-1 RNA / CSF CD4 (Log_{10})				HIV-1 DNA / CSF CD4 (Log_{10})				HIV-1 RNA / 10^6 PBMC CD4 (Log_{10})				HIV-1 DNA / 10^6 PBMC CD4 (Log_{10})			
Predictors	$R^2/\Delta R^2$	B (SE)	β	p	$R^2/\Delta R^2$	B (SE)	β	p	$R^2/\Delta R^2$	B (SE)	β	p	$R^2/\Delta R^2$	B (SE)	β	p
Step 1	0.58				0.07				0.31				0.26			
FWM NAA		-0.15 (0.04)	-0.76	0.004		-0.05 (0.06)	-0.27	0.40		-0.13 (0.06)	-0.55	0.04		-0.13 (0.06)	-0.51	0.06
Step 2	0.01				0.18				0.22				0.24			
FWM NAA		-0.14 (0.04)	-0.71	0.01		-0.01 (0.06)	-0.07	0.83		-0.11 (0.05)	-0.47	0.05		-0.11 (0.06)	-0.42	0.08
Early/Late Treated		0.06 (0.11)	0.12	0.61		0.23 (0.16)	0.47	0.17		0.32 (0.14)	0.48	0.05		0.37 (0.16)	0.50	0.04
Step 1	0.29				0.13				0.06				0.03			
PCC NAA		-0.11 (0.05)	-0.53	0.06		-0.08 (0.07)	-0.37	0.24		-0.07 (0.08)	-0.25	0.37		-0.05 (0.09)	-0.17	0.56
Step 2	0.09				0.19				0.30				0.33			
PCC NAA		-0.09 (0.05)	-0.45	0.11		-0.06 (0.07)	-0.27	0.35		-0.06 (0.06)	-0.54	0.40		-0.04 (0.07)	-0.12	0.62
Early/Late Treated		0.15 (0.13)	0.32	0.25		0.22 (0.14)	0.44	0.15		0.37 (0.15)	0.55	0.03		0.42 (0.17)	0.57	0.03
Step 1	0.07				0.06				0.03				0.07			
Caudate NAA		-0.09 (0.14)	-0.27	0.37		-0.08 (0.10)	-0.25	0.43		-0.09 (0.13)	-0.18	0.51		-0.14 (0.14)	-0.26	0.35
Step 2	0.24				0.31				0.39				0.43			
Caudate NAA		-0.12 (0.09)	-0.36	0.21		-0.12 (0.09)	-0.36	0.21		-0.15 (0.11)	-0.32	0.18		-0.21 (0.11)	-0.40	0.08
Early/Late Treated		0.24 (0.13)	0.50	0.09		0.28 (0.13)	0.57	0.06		0.42 (0.15)	0.64	0.02		0.49 (0.16)	0.67	0.008

Participants were classified as being either “early” or “late treated”, depending on whether or not they commenced suppressive ART within vs more than 12 months post-seroconversion

Table S4. ART information in the sample and CNS Penetration Effectiveness (CPE) Rank Score*

Case	arv 1	arv 2	arv3	arv 4	arv 5	arv 6	CPE rank score
1	tenofovir disoproxil fumarate	emtricitabine	rilpivirine				6
2	dolutegravir	abacavir	lamivudine	maraviroc			12
3	bictegravir	emtricitabine	tenofovir alafenamide				8
4	raltegravir	tenofovir alafenamide	emtricitabine				7
5	atazanavir	ritonavir	tenofovir alafenamide	emtricitabine			8
6	bictegravir	emtricitabine	tenofovir alafenamide				8
7	nevirapine	raltegravir	lamivudine				9
8	etravirine	raltegravir	tenofovir alafenamide	emtricitabine	darunavir	cobicistat	12
9	raltegravir	etravarine	abacavir				8
10	dolutegravir	abacavir	lamivudine	maraviroc			12
11	dolutegravir	abacavir	lamivudine				9
12	dolutegravir	abacavir	lamivudine				10
13	tenofovir alafenamide	emtricitabine	darunavir	cobicistat			7
14	darunavir	tenofovir alafenamide	emtricitabine	elvitegravir	cobicistat		9
15	dolutegravir	abacavir	lamivudine	maraviroc			12
16	bictegravir	emtricitabine	tenofovir alafenamide				8

* CPE rank Score.

Santos GMA, Locatelli I, Métral M, et al. Cross-Sectional and Cumulative Longitudinal Central Nervous System Penetration Effectiveness Scores Are Not Associated With Neurocognitive Impairment in a Well Treated Aging Human Immunodeficiency Virus-Positive Population in Switzerland. Open Forum Infectious Diseases 2019;6.
Letendre SL, Mills AM, Tashima KT, et al. ING116070: a study of the pharmacokinetics and antiviral activity of dolutegravir in cerebrospinal fluid in HIV-1-infected, antiretroviral therapy-naive subjects. Clin Infect Dis 2014;59:1032-1037.
Avedissian SN, Dyavar SR, Fox HS, Fletcher CV. Pharmacologic approaches to HIV-associated neurocognitive disorders. Curr Opin Pharmacol 2020;54:102-108.

Table S5. OM10.1 detection

Experiment setting	Prepared OM10 cell number	HIV-1 DNA analysis<br>HIV-1 copy per 1 x 10e6 cells	HIV-1 RNA analysis<br>HIV-1 copy per 1 x 10e6 cells
NO.1	1000	232	655
NO.2	200	50	114
NO.3	40	16	35
NO.4	8	5	8
NO.5	1.6	0	2
No.6	0	0	0

Table S6. CA-HIV-1 RNA transcripts analysis with or without RT reaction step

		HIV-1 RNA analysis with RT step	HIV-1 RNA analysis without RT step
**Experiment setting**	**Prepared OM10 cell number**	**HIV-1 copy per 1 x 10e6 cells (MFI) ***	**HIV-1 copy per 1 x 10e6 cells (MFI) ***
**NO.1**	**1000**	**655 (69110)**	**0 (24)**
**NO.2**	**200**	**114 (50190)**	**0 (-6)**
**NO.3**	**40**	**35 (35442)**	**0 (63)**
**NO.4**	**8**	**8 (17901)**	**0 (97)**
**NO.5**	**1.6**	**2 (5966)**	**0 (171)**
**No.6**	**0**	**0 (129)**	**0 (-60)**

* (MFI): Mean Fluorescent Intensity, less than 200 is assay background

Table S7. Evaluation of length of RT reaction time

ID	RT Reaction time (minute) *					M	SD	CV (%)
	1	2	4	6	10			
**1191**	64892	63295	67308	65593	61976	**64613**	2061	**0.032**
**1193**	64120	68888	65331	65913	69970	**66844**	2476	**0.037**
**1195**	62167	67529	71371	65952	65936	**66591**	3322	**0.050**

* (MFI): Mean Fluorescent Intensity was shown. less than 200 is assay background

M: Mean, SD: Standard Deviation, CV: Coefficient of Variation

Table S8. Evaluation of TNA extraction method for CSF cells

		RNA extraction	TNA extraction		
		RNA	TNA	DNA	RNA (TNA-DNA)
ID	Counted CD4 cells	HIV-1 copy	HIV-1 copy	HIV-1 copy	HIV-1 copy
825 Set-1	396,000	56.6	150.7	71.9	78.8
825 Set-2	99,000	32.6	114.0	28.0	86.0
825 Set-3	24,750	14.4	35.9	16.7	19.2
825 Set-4	6,188	3.5	6.4	1.7	4.7
826 Set-1	391,500	26.2	103.7	26.5	77.2
826 Set-2	97,875	9.4	69.5	11.5	58.0
826 Set-3	24,469	2.6	35.1	6.3	28.8
826 Set-4	6,117	1.5	4.1	1.1	3.1

REFERENCES

1. Antinori A, Arendt G, Becker JT, Brew BJ, Byrd DA, Cherner M, et al. Updated research nosology for HIV-associated neurocognitive disorders. Neurology. 2007;69(18):1789-99.
2. Heaton RK, Franklin DR, Ellis RJ, McCutchan JA, Letendre SL, Leblanc S, et al. HIV-associated neurocognitive disorders before and during the era of combination antiretroviral therapy: differences in rates, nature, and predictors. J Neurovirol. 2011;17(1):3-16.
3. Cysique LA, Heaton RK, Kamminga J, Lane T, Gates TM, Moore DM, et al. HIV-associated neurocognitive disorder in Australia: a case of a high-functioning and optimally treated cohort and implications for international neuroHIV research. J Neurovirol. 2014;20(3):258-68.
4. Kamminga J, Bloch M, Vincent T, Carberry A, Brew BJ, Cysique LA. Determining optimal impairment rating methodology for a new HIV-associated neurocognitive disorder screening procedure. Journal of Clinical and Experimental Neuropsychology. 2017;39(8):753-67.
5. Cysique LA, Maruff P, Darby D, Brew BJ. The assessment of cognitive function in advanced HIV-1 infection and AIDS dementia complex using a new computerised cognitive test battery. Arch Clin Neuropsychol. 2006;21(2):185-94.
6. Holdnack HA. Wechsler Test of Adult Reading: WTAR. San Antonio, TX: Psychological Corporation; 2001.
7. Henry JD, Crawford JR. The short-form version of the Depression Anxiety Stress Scales (DASS-21): construct validity and normative data in a large non-clinical sample. Br J Clin Psychol. 2005;44(Pt 2):227-39.
8. Sheehan DV, Lecrubier Y, Harnett Sheehan K, Janavs J, Weiller E, Keskiner A, et al. The validity of the Mini International Neuropsychiatric Interview (MINI) according to the SCID-P and its reliability. European Psychiatry. 1997;12(5):232-41.
9. Knobel H, Alonso J, Casado JL, Collazos J, González J, Ruiz I, et al. Validation of a simplified medication adherence questionnaire in a large cohort of HIV-infected patients: the GEEMA Study. Aids. 2002;16(4):605-13.
10. Lawton MP, Brody EM. Assessment of older people: self-maintaining and instrumental activities of daily living. Gerontologist. 1969;9(3):179-86.
11. Carey CL, Woods SP, Gonzalez R, Conover E, Marcotte TD, Grant I, et al. Predictive validity of global deficit scores in detecting neuropsychological impairment in HIV infection. J Clin Exp Neuropsychol. 2004;26(3):307-19.
12. CogState. Cogstate paediatric and adult normative data. Melbourne, Australia: Cogstate Ltd.; 2018.
13. Heaton RK, Miller SW, Taylor MJ, Grant I. Revised comprehensive norms for an expanded Halstead-Reitan Battery: Demographically adjusted neuropsychological norms for African American and Caucasian Adults Scoring Program. Odessa, FL: Psychological Assessment Resources; 2004.
14. Psychological Corporation. WAIS-III—WMS-III—WIAT-II Scoring Assistant. San Antonio, TX: Psychological Corporation; 2001.
15. Brandt J, Benedict RH. Hopkins Verbal Learning Test-Revised: Professional manual. Odessa, FL: Psychological Assessment Resources.; 2001.

16. Kongs SK, Thompson LL, Iverson GL, Heaton RK. Wisconsin Card Sorting Test-64 Card Version: Professional Manual. Odessa, FL: Psychological Assessment Resources.; 2000.
17. Norman MA, Moore DJ, Taylor M, Franklin D, Jr., Cysique L, Ake C, et al. Demographically corrected norms for African Americans and Caucasians on the Hopkins Verbal Learning Test-Revised, Brief Visuospatial Memory Test-Revised, Stroop Color and Word Test, and Wisconsin Card Sorting Test 64-Card Version. J Clin Exp Neuropsychol. 2011;33(7):793-804.
18. Suzuki K, Hu J-Y, Ishida T, Lin Y, Minote S, Liu Z, et al. Development of an Ultrasensitive HIV-1 DNA Detection Assay Based on an Automated πCode End-Point PCR System. J AIDS HIV Treat. 2019;1(2):69-88.
19. Suzuki K, Levert A, Yeung J, Starr M, Cameron J, Williams R, et al. HIV-1 viral blips are associated with repeated and increasingly high levels of cell-associated HIV-1 RNA transcriptional activity. AIDS. 2021.
20. Wong ML, Medrano JF. Real-time PCR for mRNA quantitation. Biotechniques. 2005;39(1):75-85.
21. Bustin SA, Nolan T. Pitfalls of quantitative real-time reverse-transcription polymerase chain reaction. J Biomol Tech. 2004;15(3):155-66.
22. Zeh C, Ndiege K, Inzaule S, Achieng R, Williamson J, Chih-Wei Chang J, et al. Evaluation of the performance of Abbott m2000 and Roche COBAS Ampliprep/COBAS Taqman assays for HIV-1 viral load determination using dried blood spots and dried plasma spots in Kenya. PLoS One. 2017;12(6):e0179316.
23. Jagodzinski LL, Manak MM, Hack HR, Liu Y, Malia JA, Freeman J, et al. Impact of Early Antiretroviral Therapy on Detection of Cell-Associated HIV-1 Nucleic Acid in Blood by the Roche Cobas TaqMan Test. Journal of clinical microbiology. 2019;57(5).
24. Tuaillon E, Kania D, Pisoni A, Bollore K, Taieb F, Ontsira Ngoyi EN, et al. Dried Blood Spot Tests for the Diagnosis and Therapeutic Monitoring of HIV and Viral Hepatitis B and C. Front Microbiol. 2020;11:373.
25. Sabi I, Mahiga H, Mgaya J, Geisenberger O, Kastner S, Olomi W, et al. Accuracy and Operational Characteristics of Xpert Human Immunodeficiency Virus Point-of-Care Testing at Birth and Until Week 6 in Human Immunodeficiency Virus-exposed Neonates in Tanzania. Clin Infect Dis. 2019;68(4):615-22.
